# Amplification of Dissymmetry Factors in π-Extended [7]- and [9]Helicenes

**DOI:** 10.1021/jacs.0c13197

**Published:** 2021-03-18

**Authors:** Zijie Qiu, Cheng-Wei Ju, Lucas Frédéric, Yunbin Hu, Dieter Schollmeyer, Grégory Pieters, Klaus Müllen, Akimitsu Narita

**Affiliations:** † Max Planck Institute for Polymer Research, Ackermannweg 10, 55128 Mainz, Germany; ‡ Department of Chemistry, University of Cologne, Greinstr. 4-6, 50939 Cologne, Germany; § Université Paris-Saclay, CEA, INRAE, Département Médicaments et Technologies pour la Santé (DMTS), SCBM, F-91191, Gif-sur-Yvette, France; ∥ Department of Organic and Polymer Chemistry, College of Chemistry and Chemical Engineering, Central South University, Changsha, Hunan 410083, People’s Republic of China; ⊥ Institute of Organic Chemistry, Johannes Gutenberg-University Mainz, Duesbergweg 10-14, 55099 Mainz, Germany; # Organic and Carbon Nanomaterials Unit, Okinawa Institute of Science and Technology Graduate University, 1919-1 Tancha, Onna-son, Kunigami-gun, Okinawa 904-0495, Japan

## Abstract

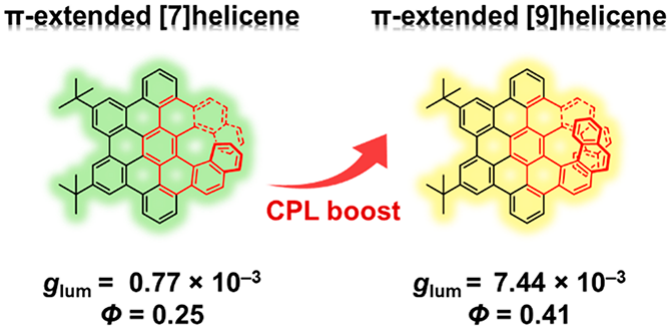

π-Extended helicenes constitute an important class of polycyclic aromatic hydrocarbons with intrinsic chirality. Herein, we report the syntheses of π-extended [7]helicene **4** and π-extended [9]helicene **6** through regioselective cyclodehydrogenation in high yields, where a “prefusion” strategy plays a key role in preventing undesirable aryl rearrangements. The unique helical structures are unambiguously confirmed by X-ray crystal structure analysis. Compared to the parent pristine [7]helicene and [9]helicene, these novel π-extended helicenes display significantly improved photophysical properties, with a quantum yield of 0.41 for **6**. After optical resolution by chiral high-performance liquid chromatography, the chiroptical properties of enantiomers **4**-*P*/*M* and **6**-*P*/*M* are investigated, revealing that the small variation in helical length from [7] to [9] can cause an approximately 10-fold increase in the dissymmetry factors. The circularly polarized luminescence brightness of **6** reaches 12.6 M^–1^ cm^–1^ as one of the highest among carbohelicenes.

## Introduction

Carbohelicenes constitute a unique class of polycyclic aromatic hydrocarbons with benzene rings that are angularly annulated in the *ortho-*configuration. The helical structures lead to intrinsic chirality and allow applications in asymmetric catalysis, nonlinear optics, and molecular machines.^1,2^ Theoretical studies have shown that the dissymmetry factor (*g*) of single-stranded [*n*]carbohelicenes increases with the helical length *n*.^3^ Therefore, tremendous efforts have been made to synthesize higher [*n*]helicenes since the first report of [6]helicene by Newman and Lednicer in 1956.^4−7^ To date, the longest carbohelicene reported is [16]helicene, which was synthesized by Fujita and co-workers in 2015.^8^ The low yield of the final photocyclization step (7%), however, hinders a further increase of the helical length by this approach.

Another research direction in helicene chemistry is the lateral extension of π-conjugated systems.^9−20^ With more extensive conjugation, π-extended helicenes can be regarded as nanosolenoids and are predicted to possess intriguing electronic, magnetic, and spin properties.^21−23^ In addition, their fascinating chiroptical features, such as circular dichroism (CD) and circularly polarized luminescence (CPL), have been intensively studied and are valuable for circularly polarized organic light-emitting diodes and bioimaging applications.^24−27^

**Scheme 1:**
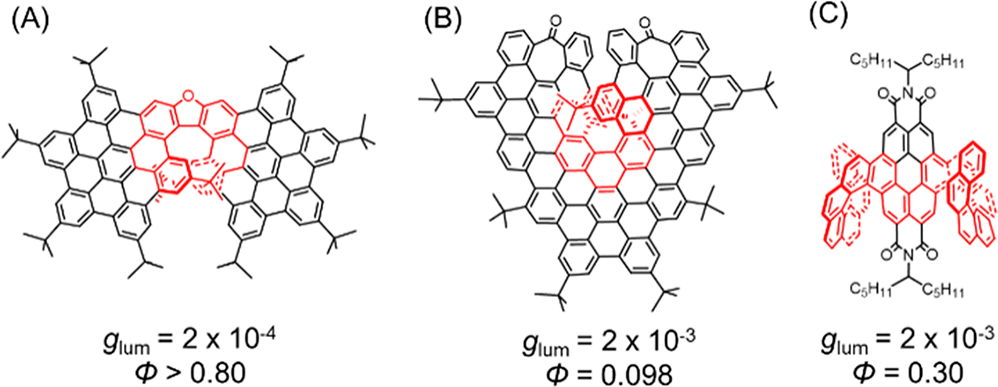
π-Extended Helicenes and Their CPL Properties

An ideal CPL emitter should possess both a high emission quantum yield (Φ) and a large luminescence dissymmetry factor (*g*_lum_), but these properties are often difficult to achieve simultaneously. One rare cylindrical molecule with *D*_4_ symmetry was reported to possess a Φ of 0.80 and an exceptional |*g*_lum_| of 0.152 by Isobe et al.^28^ Hexa-*peri*-hexabenzocoronene (HBC) and perylene diimide (PDI) have been commonly used as the skeletons for π-extension. However, the potential of such π-extended helicenes as CPL emitters has not been well explored. For example, an excellent Φ (>0.80) was achieved by a HBC-fused oxa[7]superhelicene, but its *g*_lum_ was only 2 × 10^–4^ (Scheme 1A);^10,29^ a moderate *g*_lum_ (2 × 10^–3^) and a low Φ (0.098) were reported for another HBC-based undecabenzo[7]superhelicene (Scheme 1B);^9,23^ and in a series of PDI-embedding double [8]helicenes, only moderate values of *g*_lum_ (up to 2 × 10^–3^) and Φ (up to 0.30) were observed (Scheme 1C).^19^ After the initial submission of this manuscript, Santoro, Schuster, Nuckolls et al. reported amplified CD signals by extending the helical length, but did not study the CPL performance.^27^ Therefore, the design and synthesis of π-extended helicenes with a good balance between fluorescence performance and dissymmetry factors are highly desired.

**Scheme 2:**
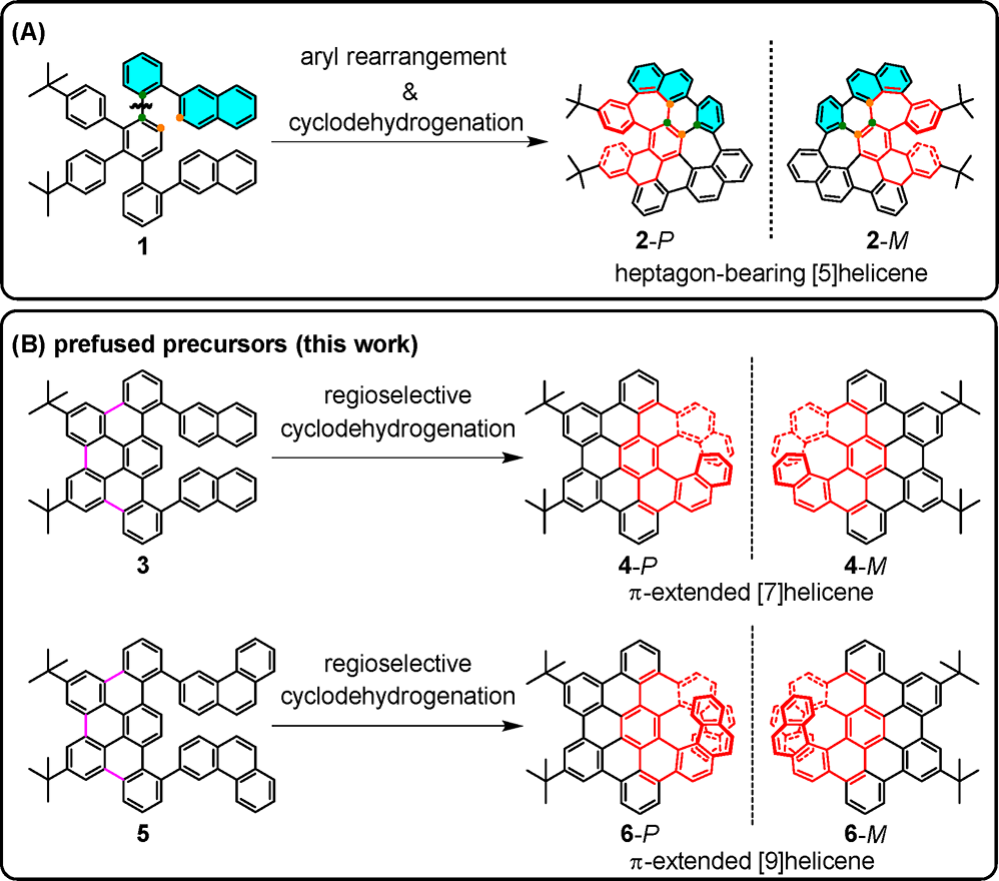
Illustration of the Prefusion Strategy To Prevent Aryl Rearrangement and Achieve the Desired π-Extended Helicenes **4** and **6**

In this study, we targeted a series of π-extended [*n*]helicenes with various helical lengths *n*. The tribenzo[*fg*,*ij*,*rst*]pentaphene, a segment of HBC, is selected as the π-extension motif, which is expected to inherit the merits of HBC in terms of optoelectronic and photophysical properties.^30^ In our first attempt to synthesize π-extended [7]helicene **4** from precursor **1**, heptagon-bearing [5]helicene **2** was selectively obtained due to unexpected aryl rearrangement during cyclodehydrogenation (Scheme 2A).^31^ Computational studies of the reaction mechanism indicated that the rearrangement occurred in the first step of dehydrogenation and was favored over direct C–C bond formation for **4**. To prevent this undesired yet highly efficient aryl rearrangement, we herein adopted a new strategy that employs precursors **3** and **5** by prefusing the tetraphenylbenzene moiety (Scheme 2B). Targeted π-extended helicenes **4** and **6** were thus successfully obtained by regioselective cyclodehydrogenation in high yields. The helical structures of **4** and **6** were confirmed by NMR spectroscopy and X-ray crystallography. Their high isomerization barriers (>40 kcal/mol) enabled the separation of enantiomers **4**-*P*/*M* and **6**-*P*/*M* by chiral high-performance liquid chromatography (HPLC). Intriguingly, the combination of the elongated helical length and extended π-conjugation empower **6** as a promising CPL emitter with a *Φ*_*f*_ of 0.41 and a *g*_lum_ of 7.4 × 10^–3^, distinguishing it from π-extended carbohelicenes in the literature.

## Results and Discussion

**Figure 1:**
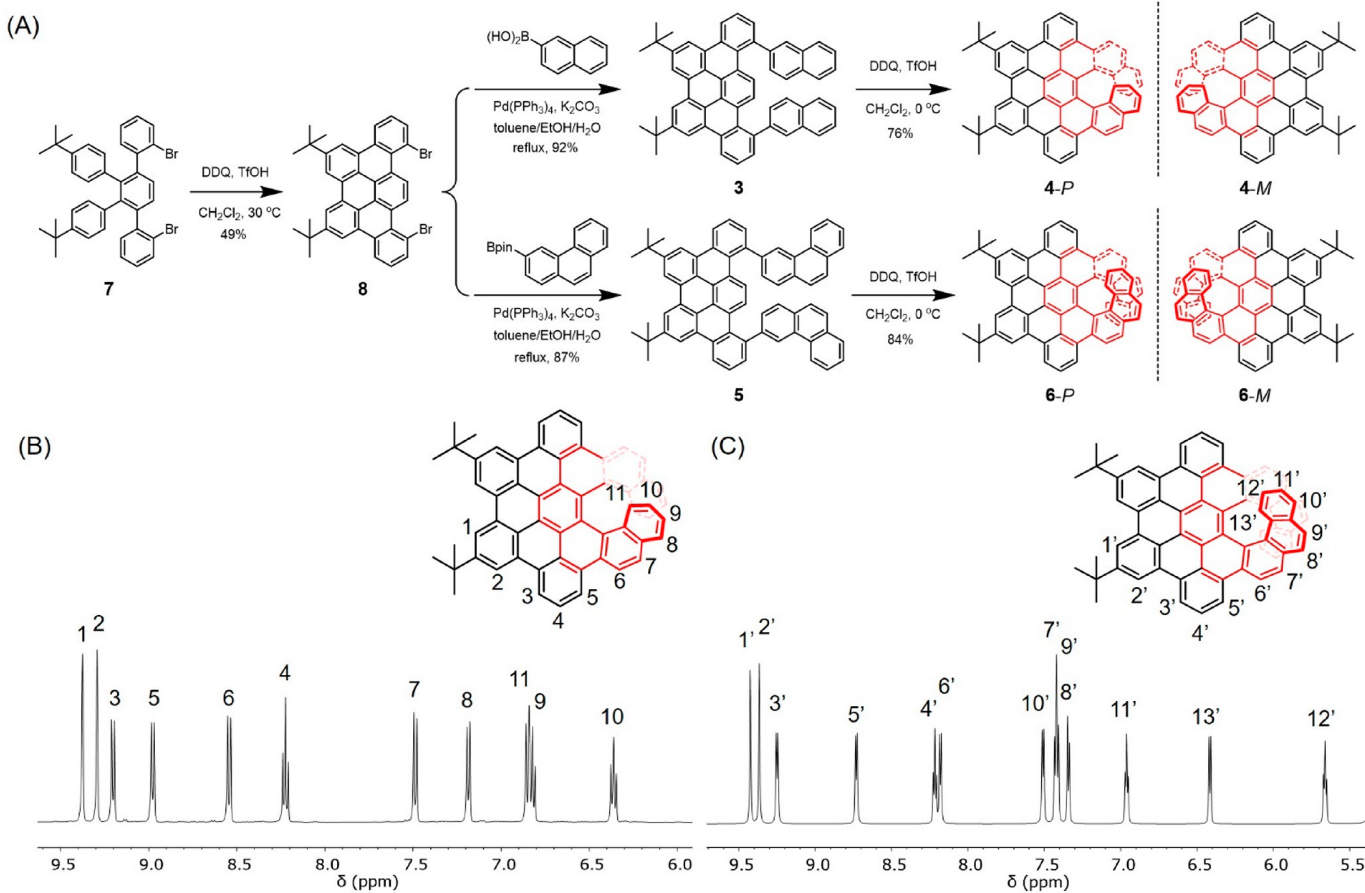
(A) Synthetic route toward **4** and **6**. (B and C) Aromatic regions of the ^1^H NMR spectra of **4** and **6** with peak assignments.

As depicted in Figure 1A, the syntheses of π-extended helicenes **4** and **6** started from dibromo-functionalized 1,2,3,4-tetraphenyl benzene **7**, which was reported in a previous paper.^31^ Compound **7** was treated with 2,3-dichloro-5,6-dicyano-1,4-benzoquinone (DDQ) and trifluoromethanesulfonic acid (TfOH) in dry dichloroethane at 30 °C under nitrogen to produce dibromo tribenzo[*fg*,*ij*,*rst*]pentaphene **8** as the prefused building block in 49% yield. Compound **8** was then coupled to 2-naphthyl groups by the Suzuki reaction to yield precursor **3**. Compared to those in precursor **1**, the phenyl rings in **3** were fully fused and thus incorporated into the polycyclic lattice, leaving only the naphthyl groups for the subsequent Scholl reaction. The final cyclodehydrogenation using DDQ and TfOH proceeded regioselectively at 0 °C, affording the desired π-extended [7]helicene **4** as a yellow solid in 76% yield. Similarly, precursor **5** functionalized with phenanthryl units was synthesized from **8**. The subsequent regioselective cyclodehydrogenation of **5** resulted in π-extended [9]helicene **6** in a high yield of 84%. The regioselective cyclodehydrogenation of **3** and **5** could also be achieved in similar yields (72% and 79%, respectively) by using FeCl_3_ as oxidant at room temperature, but no reaction was observed in oxidative photocyclization by iodine without heating. Notably, the conditions of highly regioselective Scholl reaction (DDQ/FeCl_3_) of the phenanthryl units in this work are much milder than the previously reported oxidative photocyclization (100 °C for 24 h).^32^

The chemical structures of π-extended helicenes **4** and **6** were fully characterized by standard spectroscopic techniques. In high-resolution matrix-assisted laser desorption/ionization-time-of-flight mass spectrometry (MALDI-TOF MS), **4** and **6** displayed strong signals at *m*/*z* = 736.3110 and 836.3443, respectively, with isotopic distribution patterns consistent with the calculated spectra (Figures S8 and S16). With the aid of ^1^H–^1^H correlation spectroscopy measurements, all proton peaks of **4** and **6** in the aromatic region were assigned (Figure 1B and C). Notably, the proton signals corresponding to the end of the helices (peaks 9, 10, and 11 in **4**; peaks 11′, 12′, and 13’ in **6**) exhibited pronounced upfield chemical shifts (δ = 5.57–7.00 ppm) due to the shielding effects induced by spatial overlap with other benzene rings.

**Figure 2:**
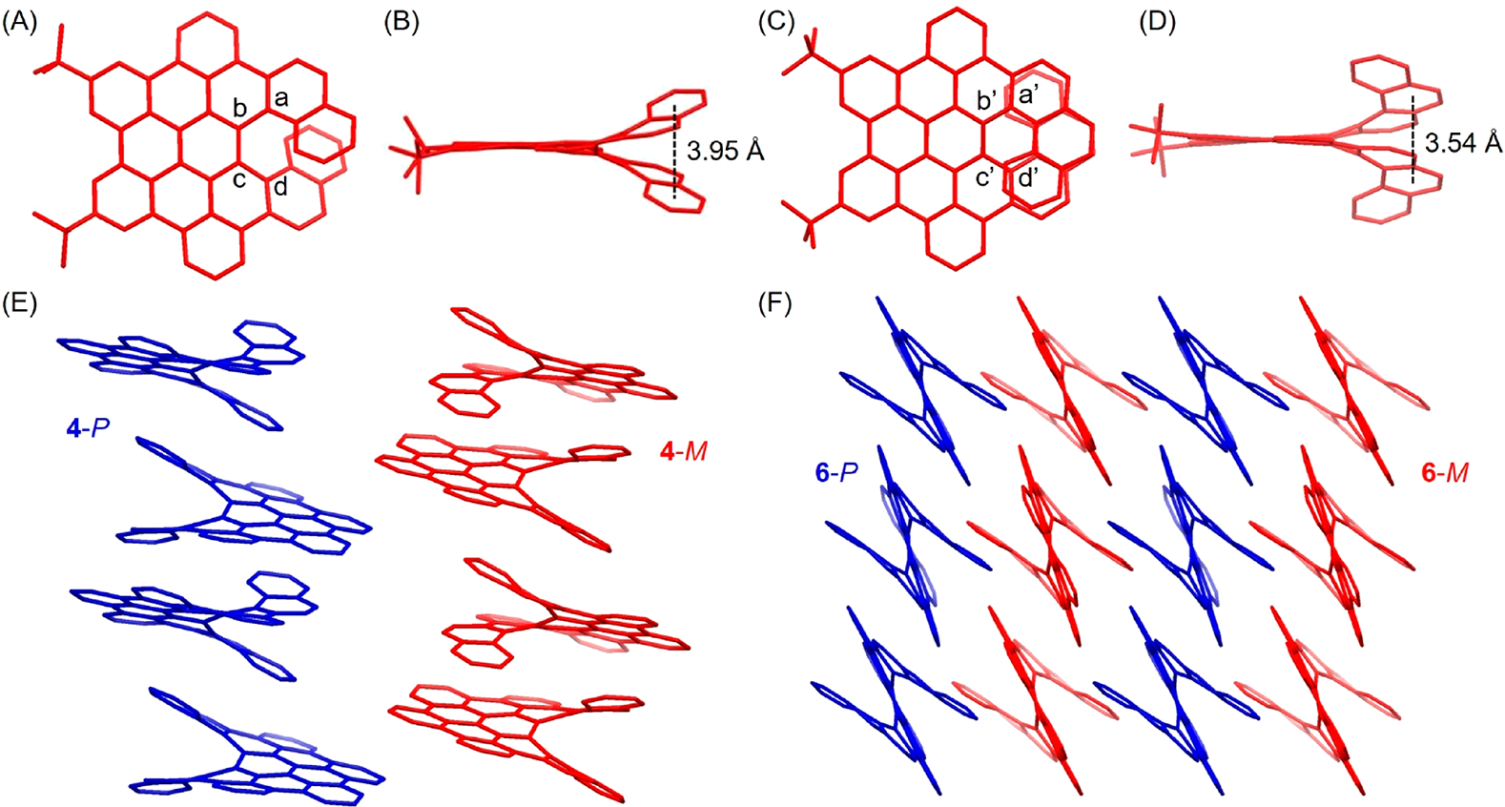
Single-crystal structures of (A and B) **4**-*M* and (C and D) **6**-*M*. (E and F) Molecular packing of **4** and **6**. All hydrogen atoms and the *tert*-butyl groups in (E and F) are omitted for clarity. The *P*- and *M*-enantiomers are highlighted in blue and red, respectively.

Single crystals of precursor **3** as well as π-extended helicenes **4** and **6** were grown by slow diffusion of ethanol vapor into their chloroform solutions (Figures 2 and S17). The helical structures of **4** and **6** were thus confirmed by X-ray diffraction. Due to the rigidification provided by the tribenzo[*fg*,*ij*,*rst*]pentaphene subunits, the torsion angles in the helices were similar, with values of 20.6° for **4**-*M* (atoms a–b–c–d) and 20.9° for **6**-*M* (atoms a′–b′–c′–d′), as depicted in Figure 2A and 2C. The helical pitch, which was determined from the centroid–centroid distance of the overlapping benzene rings (Figure 2B and 2D), was 3.95 and 3.54 Å in **4** and **6**, respectively. These lengths are slightly larger than the values for parent [7]helicene **9** (3.87 Å; CCDC: 852537) and [9]helicene **10** (3.52 Å; CCDC: 1051158) reported in the literature (the chemical structures of **9** and **10** are shown in Scheme S1).^8,33^*P*/*M* enantiomer pairs were identified in the molecular packing, where enantiomers with the same chirality (*P* or *M*) are packed in a columnar fashion in both **4** and **6** (Figure 2E and F). However, pronounced intermolecular π–π interactions were suppressed by the twisted helical substructure.

**Figure 3:**
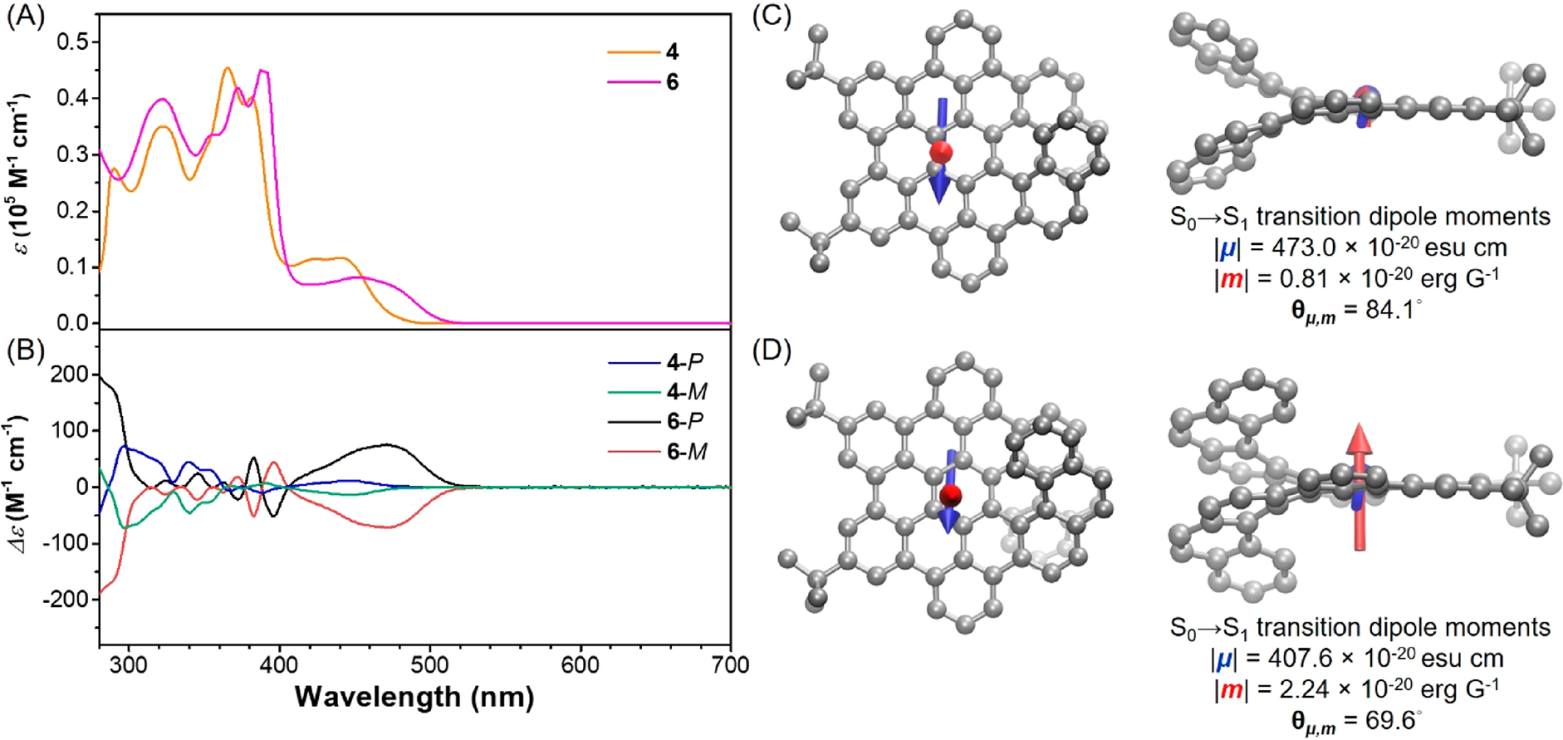
(A) Absorption spectra and (B) CD spectra of **4** and **6** in THF solutions. Solution concentration: 10^–5^ M. (C and D) Transition dipole moments of (C) **4**-*P* and (D) **6**-*P* for the S_0_ → S_1_ transition. The electric transition dipole moments (μ) are shown in blue, and the magnetic transition dipole moments (***m***) are shown in red. The length of the ***m*** vector is amplified 200 times for clarity.

The absorption and emission spectra of **4** and **6** in THF solutions were investigated and exhibited similar shapes (Figures 3A and S18). The absorption maximum (λ_abs_) of **4** was at 441 nm, and its emission peak (λ_em_) was centered at 495 nm. Because of its increased helical length *n*, **6** possesses greater π-conjugation than **4**, as supported by its red-shifted absorption and emission bands (λ_abs_ = 452 nm and λ_em_ = 528 nm). Interestingly, **4** and **6** emitted strong greenish fluorescence with Φ of 0.25 and 0.41, respectively, whereas **9** and **10** displayed much lower values (<0.02).^34,35^ This clearly demonstrates the added value of the π-extension in terms of photophysical properties. The transient PL spectra revealed an average lifetime of 16.2 ns for **4** and 8.8 ns for **6**, confirming the prompt fluorescence nature of their emission (Figure S19). Since similar nonradiative rates (*k*_nr_) were observed for **4** and **6** (4.6 × 10^7^ s^–1^ and 6.8 × 10^7^ s^–1^, respectively), the higher fluorescence Φ of **6** can be attributed to the increase in the radiative rate constant (*k*_r_ = 1.5 × 10^7^ s^–1^ for **4** and *k*_r_ = 4.5 × 10^7^ s^–1^ for **6**). In addition, these π-extended helicenes were also emissive in the solid state (Φ = 0.17 and 0.34 for **4** and **6**, respectively) with red-shifted bands (Figure S18) as a result of their nonplanar structures and thus suppressed intermolecular π–π stacking. By means of time-dependent density functional theory (TD-DFT) calculations, the first absorption peaks of **4** and **6** were assigned to the HOMO → LOMO transitions (H → L), where the electron cloud was distributed throughout the whole molecule (Figure S22). The photophysical properties and calculated major transitions of **4** and **6** are summarized in Tables S1–S3.

To investigate the *P*/*M* racemization barriers of **4** and **6**, DFT calculations were performed to identify the transition states with the highest Gibbs free energy, in which the terminal benzene rings in the helix were oriented in a face-to-face pattern (Figure S24). Accordingly, the *P*/*M* isomerization barriers of **4** and **6** were calculated to be 42.4 and 41.6 kcal/mol, respectively. These values are close to those reported for **9** and **10**,^36^ indicating that π-extension barely affected the rigidity of the helical backbones. Such high *P*/*M* isomerization barriers are marked by the high thermal stability of their enantiomers. No racemization was observed when the solutions of **4**-*M* and **6**-*M* were heated at 150 °C for 60 min (Figure S21).

Due to the high isomerization barriers, the enantiomers of **4** and **6** could be completely resolved by HPLC with a Daicel Chiralpak IE column (Figure S20). The CD spectra of isolated enantiomers **4**-*P*/*M* and **6**-*P*/*M* in THF solutions (10^–5^ M) were measured. Upon comparing the experimental and DFT-simulated CD spectra, the absolute configurations in the first and second fractions of the chiral HPLC analysis were assigned as the *P*- and *M*-enantiomers, respectively, for both **4** and **6**. Interestingly, because of the increase in the helical length *n* from 7 to 9, π-extended [9]helicene **6** exhibited a much higher Δε than **4** in the long-wavelength region (Figure 3B). From the UV–vis spectra, the absorption dissymmetry factors (*g*_abs_ = Δε/ε)^37^ of **4**-*P* and **6**-*P* at their absorption maximum peaks were calculated to be 1.24 × 10^–3^ and 10.58 × 10^–3^, respectively (Table 1). The dramatically higher value of *g*_abs_ for **6** was also supported by the simulated CD spectra (Figure S23A). For comparison, the CD spectra of non-π-extended helicenes **9** and **10** were also simulated by TD-DFT at the same level of theory. Unlike those of π-extended helicenes **4** and **6**, the CD signal intensities of **9** and **10** were not substantially affected by increasing the helical length *n* (Figure S23B).^3^ According to the absorption peak assignment discussed above, the first peak in the CD spectra originates from the chirality of the whole molecule for both **4** and **6**. Consequently, the drastic changes in the dissymmetry factors of our π-extended helicenes result from the combined effect of lateral and helical extensions.

**Table 1:** Summary of the Chiroptical Properties of **4**-*P* and **6**-*P*

	CD^a^				S_0_→S_1_ transition^b^				CPL^a^		
	λ (nm)	Δε (M^–1^ cm^–1^)	*Ε* (M^–1^ cm^–1^)	*g*_abs_ (10^–3^)	|μ| (10^–20^ esu cm)	**|m|** (10^–20^ erg G^–1^)	θ (deg)	*g*_cal_ (10^–3^)	λ_em_ (nm)	*g*_lum_ (10^–3^)	*B*_CPL_ (M^–1^ cm^–1^)
**4**-*P*	446	13.9	11 255	1.24	469.2	0.81	84.1	0.71	486	0.77	1.1
**6**-*P*	471	75.2	7108	10.58	407.0	2.24	69.6	7.60	532	7.44	12.6

a Measured in a dilute THF solution. Concentration: 10^–5^ M.

b Calculated by TD-DFT at the B3LYP/6-311G (d,p) level.

According to theory, *g*_abs_ can be determined by the following equation:
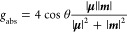
Therefore, the electronic (μ) and magnetic (***m***) transition dipole moments, as well as the angle (θ) between μ and ***m***, of **4**-*P* and **6**-*P* for their S_0_ → S_1_ transitions were determined by means of TD-DFT calculations (Table 1). For organic materials, the **|m|** value is normally much lower than the |μ| value. The above equation can thus be simplified as *g*_abs_ = 4 cos θ **|m|**/|μ|. The higher **|m|**, lower |μ|, and larger cos θ of **6** than of **4** all lead to an increase in the calculated absorption dissymmetry factor (*g*_cal_) by a factor of 10 with respect to that of **4**, consistent with the trend observed experimentally.

**Figure 4:**
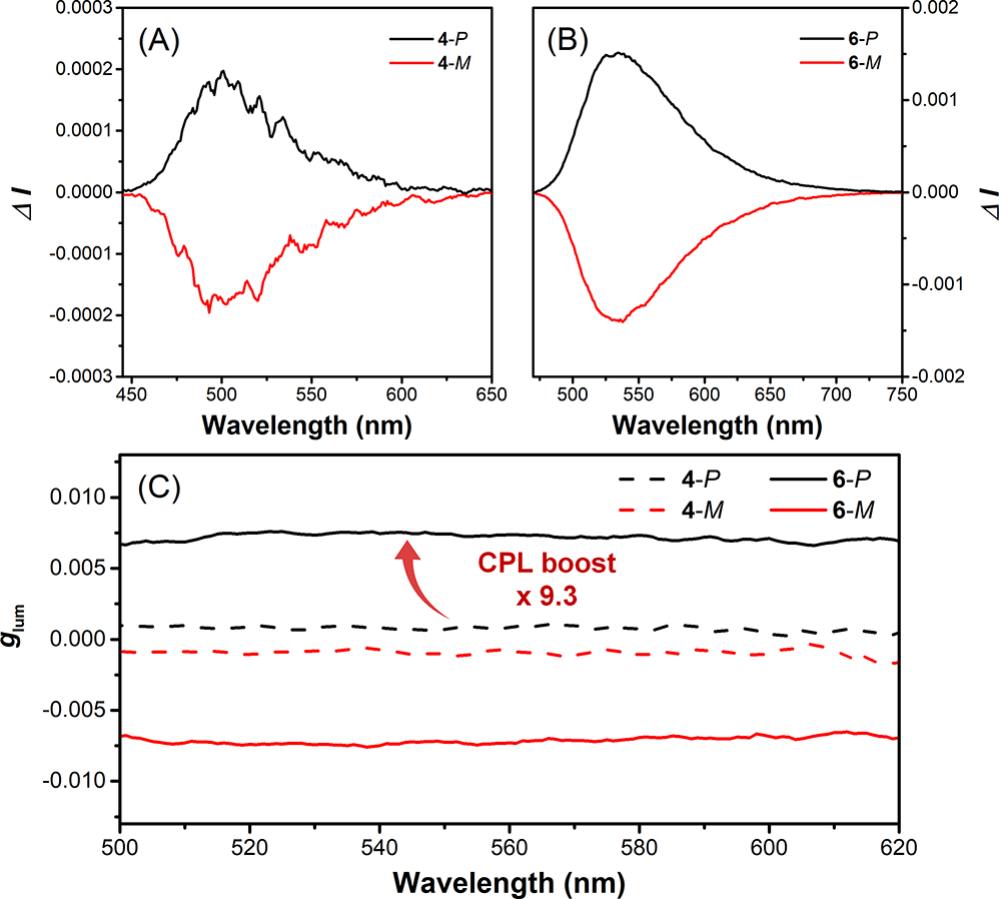
(A and B) CPL emission spectra and (C) luminescence dissymmetry factors of **4**-*P*/*M* and **6**-*P*/*M* in THF. Concentration: 10^–5^ M. Excitation: 380 nm for **4** and 425 nm for **6**.

Subsequently, the CPL spectra of **4**-*P*/*M* and **6**-*P*/*M* were also measured to explore the potential of these compounds as chiral emitters.^37^ Mirror images of the CPL spectra and *g*_lum_ plots were observed for the *P*- and *M*-enantiomers of both **4** and **6** (Figure 4). Similar to the CD properties, the CPL intensity (Δ*I*) and *g*_lum_ of **6** were significantly enhanced (*g*_lum, **6**-*P*_ = 7.4 × 10^–3^) with a high signal-to-noise ratio. Following the concept of fluorescence brightness, the CPL brightness (*B*_CPL_) has recently been proposed to evaluate the overall performance of CPL emitters:^38^
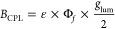
With all the necessary chiroptical results in hand, the *B*_CPL_ of **6** was calculated to be 12.6 M^–1^ cm^–1^, which is one of the highest values among all carbohelicenes reported in the literature,^38^ indicating that **6** may be an excellent emitter for CPL applications.

## Conclusion

In summary, two π-extended helicenes, **4** and **6**, were synthesized through regioselective cyclodehydrogenation in high yields. The design of prefused precursors **3** and **5** plays a key role in preventing undesirable aryl rearrangements. Studies of the chiroptical properties of these compounds have revealed the beneficial effect of their π-extension and helical subunits on their dissymmetry factors. Approximately 10-fold enhancements in *g*_abs_, *g*_lum_, and *B*_CPL_ were observed from **4** to **6**, indicating that **6** is a promising CPL emitter. More importantly, **4** and **6** can be used as model compounds for other π-extended helicenes with even higher helical lengths currently under investigation in our laboratory following the polymerization–cyclodehydrogenation approach. Because of both extended π-conjugation and stable chirality, this series of π-extended helicenes are expected to possess high potential for spin transport^39−41^ and superior inductance.^21^

## Supporting Information Available

Experimental details, characterization spectra of all synthesized compounds, single-crystal data, photophysical measurements, and computational details (PDF)

## Accession Codes

CCDC 2047540–2047542 contain the supplementary crystallographic data for this paper. These data can be obtained free of charge via www.ccdc.cam.ac.uk/data_request/cif, or by emailing data_request@ccdc.cam.ac.uk, or by contacting The Cambridge Crystallographic Data Centre, 12 Union Road, Cambridge CB2 1EZ, UK; fax: +44 1223 336033.
