# Supplementary material for: Amplification of Dissymmetry Factors in π-Extended [7]- and [9]Helicenes
Source: J Am Chem Soc. 2021 Mar 18;143(12):4661–7. doi: 10.1021/jacs.0c13197 (PMC8041289; doi:10.1021/jacs.0c13197)
Supplement: Supplementary file 1 — ja0c13197_si_001.pdf [file ja0c13197_si_001.pdf]

Supporting Information

Amplification of Dissymmetry Factors in π-Extended [7]- and [9]Helicenes

Zijie Qiu,[1] Cheng-Wei Ju,[1] Lucas Frédéric,[3] Yunbin Hu,[1,4] Dieter Schollmeyer,[5] Grégory Pieters,[*,3] Klaus Müllen,[*,1,2] Akimitsu Narita[*,1,6]

[1] Max Planck Institute for Polymer Research, Ackermannweg 10, 55128 Mainz, Germany

[2] Department of Chemistry, University of Cologne, Greinstr. 4-6, 50939 Cologne, Germany

[3] Université Paris-Saclay, CEA, INRAE, Département Médicaments et Technologies pour la Santé (DMTS), SCBM, F-91191, Gif-sur-Yvette, France

[4] Department of Organic and Polymer Chemistry, College of Chemistry and Chemical Engineering, Central South University, Changsha, Hunan 410083, People's Republic of China

[5] Institute of Organic Chemistry, Johannes Gutenberg-University Mainz, Duesbergweg 10-14, 55099 Mainz, Germany

[6] Organic and Carbon Nanomaterials Unit, Okinawa Institute of Science and Technology Graduate University, 1919-1 Tancha, Onna-son, Kunigami-gun, Okinawa 904-0495, Japan

Table of Contents

1. Experimental Section
2. NMR and HRMS Spectra (Figure S1-S16)
3. X-Ray Single Crystallography (Figure S17)
4. Photophysical Properties and Chiral HPLC analysis (Figure S18-S21, Scheme S1, Table S1)
5. DFT Calculations (Figure S22-S24, Table S2-S5)

1. Experimental Section

General Methods

All reactions working with air- or moisture-sensitive compounds were carried out under nitrogen atmosphere using standard Schlenk line techniques. Unless otherwise noted, all starting materials were purchased from commercial sources and used without further purification. All other reagents were used as received. Preparative column chromatography was performed on silica gel from Merck with a grain size of 0.063-0.200 mm (silica gel). Nuclear Magnetic Resonance (NMR) spectra were recorded in CD_2Cl_2 or $C_2D_2Cl_4$ on AVANCE 300 MHz, AVANCE 500 MHz, or AVANCE 850 MHz Bruker spectrometers. Abbreviations: s = singlet, d = doublet, t = triplet, q = quartet, m = multiplet, dd = doublet of doublets, br = broad signal. High-resolution mass spectrometry (HRMS) was performed on a SYNAPT G2 Si high-resolution time-of-flight mass (TOF) spectrometer (Waters Corp., Manchester, UK) by matrix-assisted laser desorption/ionization (MALDI). UV-vis absorption spectra were recorded on a Perkin-Elmer Lambda 900 spectrophotometer using a 10 mm quartz cell. Photoluminescence spectra were recorded on an Edinburgh Instruments FS5 fluorescence spectrometer. Solution quantum yields and solid-state quantum yields were determined on an Edinburgh Instruments FS5 fluorescence spectrometer with a calibrated integrating sphere system. To reduce the fluctuation in the excitation intensity, the lamp was kept on for 1 hour prior to the experiment. Time-resolved photoluminescence (TRPL) measurements were performed on an Edinburgh Instruments FS5 fluorescence spectrometer with Edinburgh pulsed laser (450 nm), based on time correlated single-photon counting (TCSPC) technique. Chiral high-performance liquid chromatography (HPLC) was implemented on a Daicel Chiralpak IE column. The circular dichroism (CD) spectra were collected on JASCO J-1500 circular dichroism spectrometer at 298 K. The circularly polarized luminescence (CPL) measurements were performed using a JASCO CPL-300 at room temperature with 10 × 10 mm cells. Data pitch was set at 1 nm, scanning speed was set at 100 nm/min and spectra displayed are mean values of 10 accumulations. Excitation wavelength and instrument parameters were adapted for every sample as follow: Compounds **4**-*P*/*M*: excitation wavelength: 380 nm, measurement range: 650-445 nm, excitation slit width: 1000 um, emission slit width: 1000 um, HT voltage (photomultiplier): 650 V; Compounds **6**-*P*/*M*: excitation wavelength: 425 nm, measurement range: 750-470 nm, excitation slit width: 1000 um, emission slit width: 1000 um, HT voltage (photomultiplier): 670 V.

Synthesis

2-Bromo-4'-(2-bromophenyl)-4''-(tert-butyl)-3'-(4-*tert*-butylphenyl)-1,1':2',1''-terphenyl (**7**) was synthesized according to the previous report.[1]

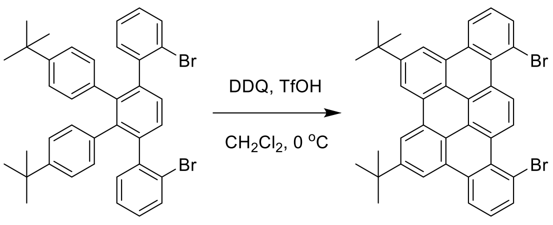

7 **8**

1,14-Dibromo-6,9-di-*tert*-butyltribenzo[*fg,ij,rst*]pentaphene (8): Under argon atmosphere, compound **7** (100 mg, 0.153 mmol) and 2,3-dichloro-5,6-dicyano-1,4-benzoquinone (DDQ) (209 mg, 0.921 mmol, 6 equiv.) were dissolved in 10 mL of dry dichloromethane (DCM). The resulting solution was cooled to 0 °C and 0.1 mL triflic acid (TfOH) was then added dropwise. The reaction mixture was then heated to 30 °C and stirred for 3 hours. After the full consumption of compound **7** as monitored by thin layer chromatography (TLC), the reaction mixture was successively neutralized by triethylamine, diluted with DCM, and washed three times with brine. The organic phase was dried over Mg_2SO_4 and evaporated to dryness. The residue was purified by silica gel column chromatography (hexane:DCM = 10:1 as eluent), affording 48 mg of compound **8** as a light yellow solid in 49% yield. ^{1}H NMR (300 MHz, dichloromethane-d_2): δ 9.30 (s, 2H), 8.44 (s, 2H), 8.29 (s, 2H), 8.23 (d, J = 8.1 Hz, 2H), 7.44 (d, J = 7.5 Hz, 2H), 6.92 (t, J = 7.8 Hz, 2H), 1.04 (s, 18H). ^{13}C NMR (75 MHz, dichloromethane-d_2): δ 149.34, 135.03, 133.55, 129.30, 129.08, 127.59, 123.59, 123.31, 122.98, 122.34, 120.58, 119.55, 119.24, 35.46, 31.73. HRMS (MALDI-TOF) *m/z*: Calcd for $C_{38}H_{30}Br_2$: 644.0714; Found: 644.0690 (M^+).

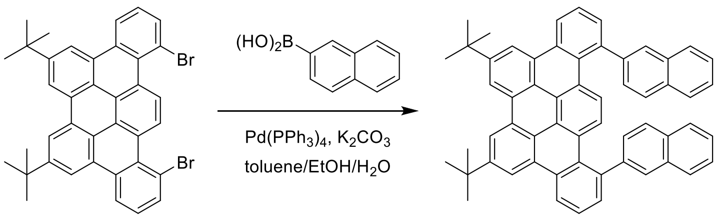

8 **3**

6,9-Di-*tert*-butyl-1,14-di(naphthalen-2-yl)tribenzo[*fg,ij,rst*]pentaphene (3): Under nitrogen atmosphere, compound **8** (100 mg, 0.155 mmol), 2-naphthylboronic acid (66 mg, 0.387 mmol, 2.5 equiv.), $Pd(PPh_3)_4$ (14 mg, 0.0124 mmol, 0.08 equiv.), and K_2CO_3 (85 mg, 0.619 mmol, 4 equiv.) were dissolved in a mixed solution of 8 mL toluene, 2 mL EtOH, and 2 mL H_2O.

Afterwards, the solution was heated to reflux overnight with stirring. After the reaction was completed as monitored by TLC, the solution was extracted with dichloromethane for three times. The combined organic phases were dried over magnesium sulfate. After the solvents were removed by rotary evaporation, the residue was purified by silica gel column chromatography (hexane:DCM = 10:1 as eluent), affording the title compound (105 mg) as light yellow solid in 92% yield. ^{1}H NMR (500 MHz, tetrachloroethane-d_2, 403 K): δ 9.18 (s, 2H), 9.08 (s, 2H), 8.90 (d, J = 8.0 Hz, 2H), 7.86 (s, 2H), 7.75 (t, J = 9.5 Hz, 6H), 7.64-7.62 (m, 4H), 7.54-7.46 (m, 6H), 7.29 (d, J = 8.0 Hz, 2H), 1.79 (s, 18H). ^{13}C NMR (126 MHz, tetrachloroethane-d_2, 403 K): δ 149.42, 142.98, 141.35, 134.18, 132.55, 132.08, 130.10, 129.92, 129.36, 128.01, 127.94, 127.92, 127.24, 126.84, 126.59, 126.02, 125.65, 125.60, 124.09, 123.21, 122.80, 119.18, 119.14, 35.64, 31.94. HRMS (MALDI-TOF) m/z: Calcd for $C_{58}H_{44}$: 740.3443; Found: 740.3430 (M^+).

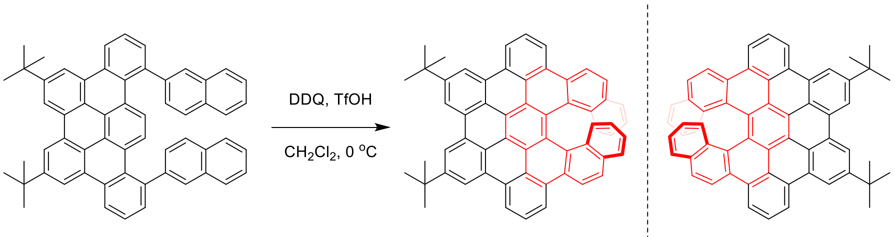

3 4-*P* 4-*M*

π-Extended [7]helicene 4 using DDQ as oxidant: Under nitrogen atmosphere, compound **3** (100 mg, 0.135 mmol) and DDQ (123 mg, 0.540 mmol, 4 equiv.) were dissolved in 30 mL of dry DCM. The resulting solution was cooled to 0 °C and 0.1 mL TfOH was then added dropwise. After stirring at 0 °C for 30 min, the reaction mixture was successively neutralized by triethylamine, diluted with DCM, and washed with brine. The organic phase was dried over magnesium sulfate and evaporated to dryness. The residue was purified by silica gel column chromatography (hexane:DCM = 10:1 as eluent), affording 75 mg of compound **4** as a yellow solid in 76% yield. ^{1}H NMR (300 MHz, dichloromethane-d_2): δ 9.38 (s, 2H), 9.30 (s, 2H), 9.21 (d, J = 7.8 Hz, 2H), 9.00 (d, J = 8.1 Hz, 2H), 8.57 (d, J = 8.7 Hz, 2H), 8.23 (t, J = 7.8 Hz, 2H), 7.51 (d, J = 8.7 Hz, 2H), 7.21 (d, J = 7.8 Hz, 2H), 6.88-6.82 (m, 4H), 6.39 (t, J = 7.8 Hz, 2H), 1.87 (s, 18H). ^{13}C NMR (75 MHz, dichloromethane-d_2): δ 149.90, 131.88, 130.94, 130.82, 130.59, 130.41, 129.74, 128.39, 138.25, 128.10, 127.20, 126.41, 125.74, 125.38, 124.87, 124.74, 123.80, 122.79, 122.54, 122.27, 121.33, 120.55, 120.41, 120.02, 119.54, 36.16, 32.18. HRMS (MALDI-TOF) m/z: Calcd for $C_{58}H_{40}$: 736.3130; Found: 736.3110 (M^+).

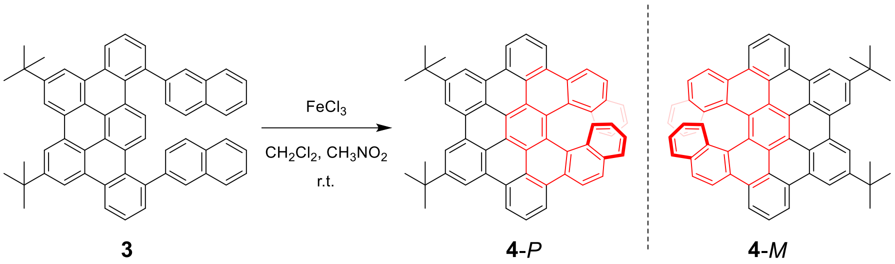

π-Extended [7]helicene 4 using $FeCl_3$ as oxidant: Under nitrogen atmosphere, compound **3** (10 mg, 0.013 mmol) was dissolved in 1 mL of unstabilized DCM and bubbled with nitrogen for 10 min. $FeCl_3$ (26 mg, 0.16 mmol, 12 equiv.) was dissolved in 0.2 mL of CH_3NO_2 and then added slowly into the solution of compound **3**. The resulting solution was kept under nitrogen and stirred at room temperature for one hour. Afterwards, the reaction mixture was quenched by methanol and washed with brine. The organic phase was dried over magnesium sulfate and evaporated to dryness. The residue was purified by silica gel column chromatography (hexane:DCM = 10:1 as eluent), affording 7.2 mg of compound **4** as a yellow solid in 72% yield. Characterization data was the same as those obtained by the DDQ conditions.

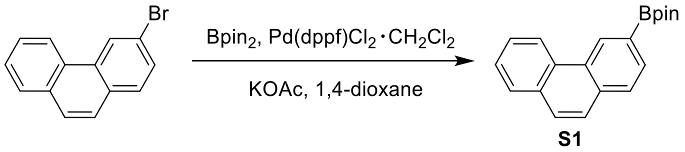

4,4,5,5-Tetramethyl-2-(phenanthren-3-yl)-1,3,2-dioxaborolane (S1): Under argon atmosphere, 3-bromophenanthrene (100 mg, 0.389 mmol), bis(pinacolato)diboron (119 mg, 0.467 mmol, 1.2 equiv.), $Pd(dppf)Cl_2 \cdot DCM$ (12.7 mg, 0.0156 mmol, 0.04 equiv.), and KOAc (145 mg, 1.478 mmol, 3.8 equiv.) were dissolved in 3 mL of 1,4-dioxane. The mixture was stirred at 80 °C overnight. After confirming the completion of the reaction by TLC, the solution was extracted with dichloromethane for three times. The combined organic phases were dried over magnesium sulfate. After the solvents were removed by rotary evaporation, the residue was purified by silica gel column chromatography (ethyl acetate/hexane = 1/10), affording 98 mg of compound **S1** as a white solid in 83% yield. ^{1}H NMR (300 MHz, dichloromethane-d_2): δ 9.18 (s, 1H), 8.83 (d, J = 8.1 Hz, 1H), 7.98-7.89 (m, 3H), 7.83-7.75 (m, 2H), 7.72-7.60 (m, 2H), 1.42 (s, 12H). ^{13}C NMR (75 MHz, dichloromethane-d_2): δ 134.38, 132.47, 132.18, 130.88, 130.34, 129.84, 128.94, 128.47, 128.10, 127.18, 127.03, 123.19, 118.57, 84.43, 25.17. HRMS (MALDI-TOF) *m*/*z*: Calcd for $C_{20}H_{21}BO_2$: 304.1635; Found: 304.1629 (M^+).

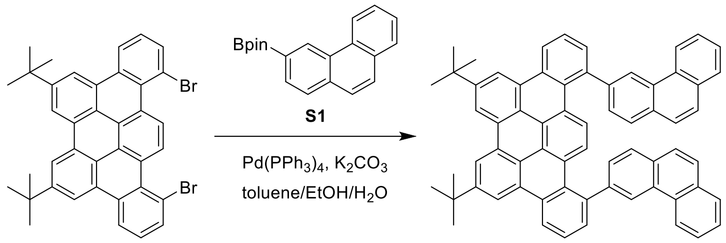

8 **5**

6,9-Di-*tert*-butyl-1,14-di(phenanthren-3-yl)tribenzo[*fg*,*ij*,*rst*]pentaphene (5): Under nitrogen atmosphere, compound **8** (100 mg, 0.155 mmol), **S1** (118 mg, 0.387 mmol, 2.5 equiv.), $Pd(PPh_3)_4$ (14.3 mg, 0.0124 mmol, 0.08 equiv.), and K_2CO_3 (85.5 mg, 0.619 mmol, 4 equiv.) were dissolved in a mix solution of 8 mL toluene, 2 mL EtOH, and 2 mL H_2O. Afterwards, the solution was heated to reflux overnight with stirring. After the reaction was completed as monitored by TLC, the solution was extracted with dichloromethane for three times. The combined organic phases were dried over magnesium sulfate. After the solvents were removed by rotary evaporation, the residue was purified by silica gel column chromatography (hexane:DCM = 10:1 as eluent), 113 mg of the title compound was obtained as light yellow solid in 87% yield. ^{1}H NMR (500 MHz, tetrachloroethane-d_2, 298 K): δ 9.22 (s, 2H), 9.11 (s, 2H), 8.93 (d, J = 8.5 Hz, 2H), 8.66 (s, 2H), 8.47 (d, J = 8.0 Hz, 2H), 7.95 (d, J = 8.0 Hz, 2H), 7.76 (d, J = 8.0 Hz, 4H), 7.65 (s, 4H), 7.65-7.50 (m, 6H), 7.34 (d, J = 7.5 Hz, 2H), 7.20 (br, 2H), 1.81 (s, 18H). ^{13}C NMR (126 MHz, tetrachloroethane-d_2, 403 K): δ 149.44, 143.68, 141.67, 132.63, 132.54, 132.33, 130.94, 130.67, 130.17, 129.94, 129.41, 128.73, 128.53, 128.04, 126.92, 126.88, 126.63, 126.61, 126.59, 126.47, 125.74, 125.25, 124.80, 124.24, 123.27, 122.87, 122.83, 119.25, 119.15, 35.67, 31.97. HRMS (MALDI-TOF) m/z: Calcd for $C_{66}H_{48}$: 840.3756; Found: 840.3734 (M^+).

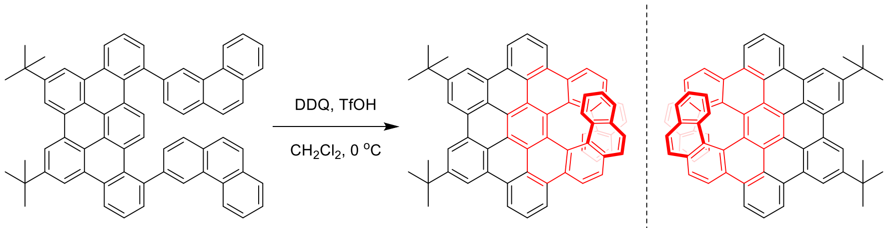

5 **6**-*P* **6**-*M*

***π*-Extended [9]helicene 6 using DDQ as oxidant:** Under argon atmosphere, compound **5** (100 mg, 0.119 mmol) and DDQ (108 mg, 0.476 mmol, 4 equiv.) were dissolved in 30 mL of dry DCM. The resulting solution was cooled to 0 °C and 0.1 mL TfOH was then added dropwise. After stirring at 0 °C for 30 min, the reaction mixture was successively neutralized by

triethylamine, diluted with DCM, and washed with brine. After extraction with DCM and separation, the organic phase was dried over magnesium sulfate and evaporated to dryness. The residue was purified by silica gel column chromatography (hexane:DCM = 5:1 as eluent), affording 84 mg of compound **6** as a yellow solid in 84% yield. ^{1}H NMR (700 MHz, dichloromethane-d_2): δ 9.39 (s, 2H), 9.33 (s, 2H), 9.21 (d, J = 7.7 Hz, 2H), 8.69 (d, J = 7.7 Hz, 2H), 8.18 (t, J = 7.4 Hz, 2H), 8.14 (d, J = 8.4 Hz, 2H), 7.47 (d, J = 7.7 Hz, 2H), 7.38 (t, J = 9.1 Hz, 4H), 7.30 (d, J = 7.7 Hz, 2H), 6.92 (t, J = 7.0 Hz, 2H), 6.38 (d, J = 8.4 Hz, 2H), 5.62 (t, J = 7.7 Hz, 2H), 1.76 (s, 18H). ^{13}C NMR (176 MHz, dichloromethane-d_2): δ 150.20, 133.07, 131.03, 130.97, 130.89, 130.83, 130.21, 128.19, 128.14, 127.55, 127.42, 127.37, 126.72, 126.53, 125.45, 125.31, 124.65, 124.24, 123.84, 123.58, 122.72, 122.44, 122.04, 121.00, 120.32, 120.21, 119.74, 36.36, 32.39. HRMS (MALDI-TOF) m/z: Calcd for $C_{66}H_{44}$: 836.3443; Found: 836.3416 (M^{+}).

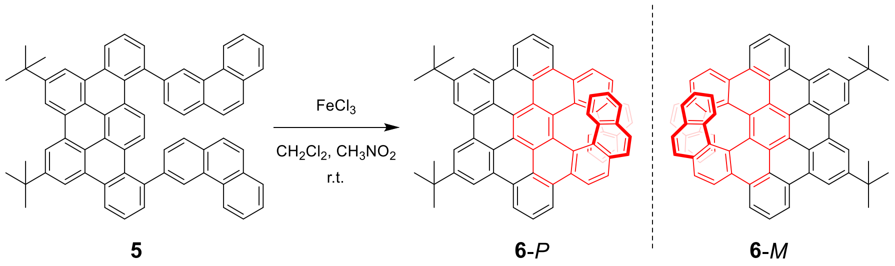

π-Extended [9]helicene 6 using $FeCl_3$ as oxidant: Under nitrogen atmosphere, compound **5** (10 mg, 0.012 mmol) was dissolved in 1 mL of unstabilized DCM and bubbled with nitrogen for 10 min. $FeCl_3$ (23 mg, 0.14 mmol, 12 equiv.) was dissolved in 0.2 mL of CH_3NO_2 and then added slowly into the solution of compound **5**. The resulting solution was kept under nitrogen and stirred at room temperature for one hour. Afterwards, the reaction mixture was quenched by methanol and washed with brine. The organic phase was dried over magnesium sulfate and evaporated to dryness. The residue was purified by silica gel column chromatography (hexane:DCM = 10:1 as eluent), affording 7.9 mg of compound **6** as a yellow solid in 79% yield. Characterization data was the same as those obtained by the DDQ conditions.

2. NMR and HRMS Spectra

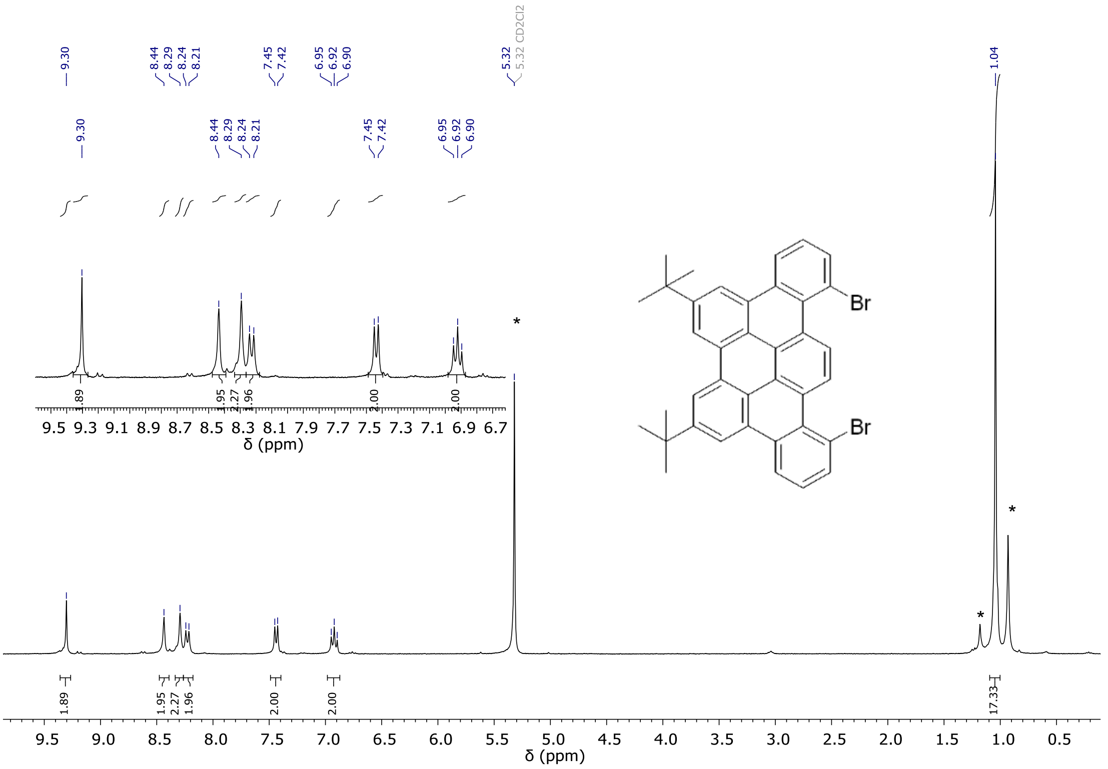

Figure S1. ^{1}H NMR spectrum of **8** in CD_2Cl_2 (300 MHz). The solvent peaks were marked with asterisks.

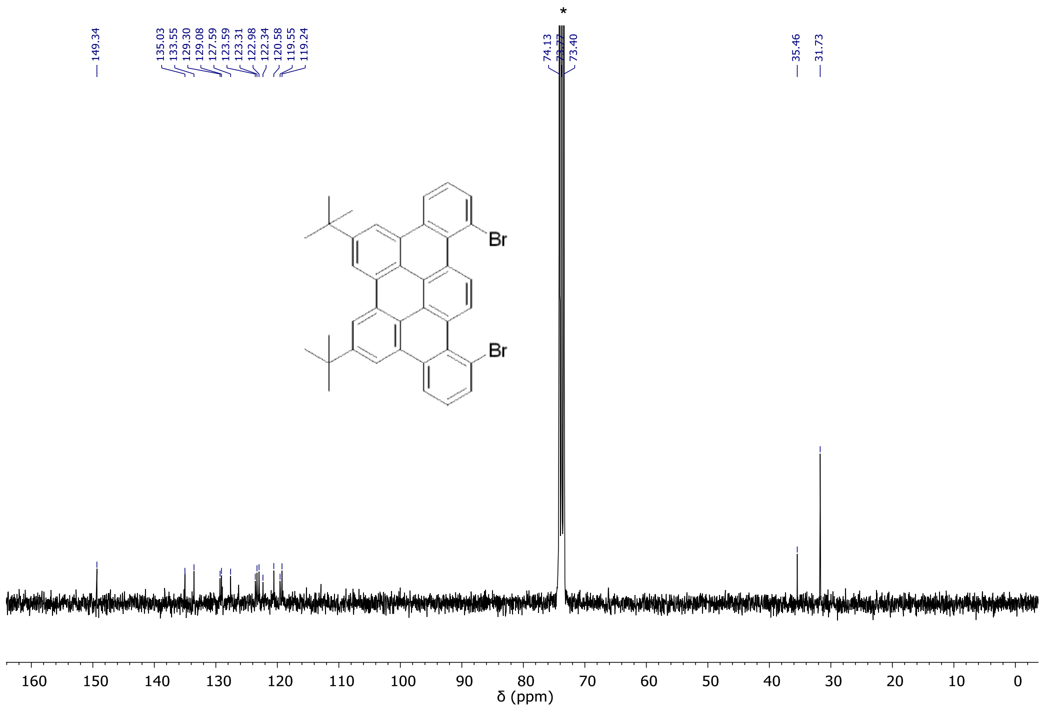

Figure S2. ^{13}C NMR spectrum of **8** in CD_2Cl_2 (75 MHz). The solvent peaks were marked with asterisks.

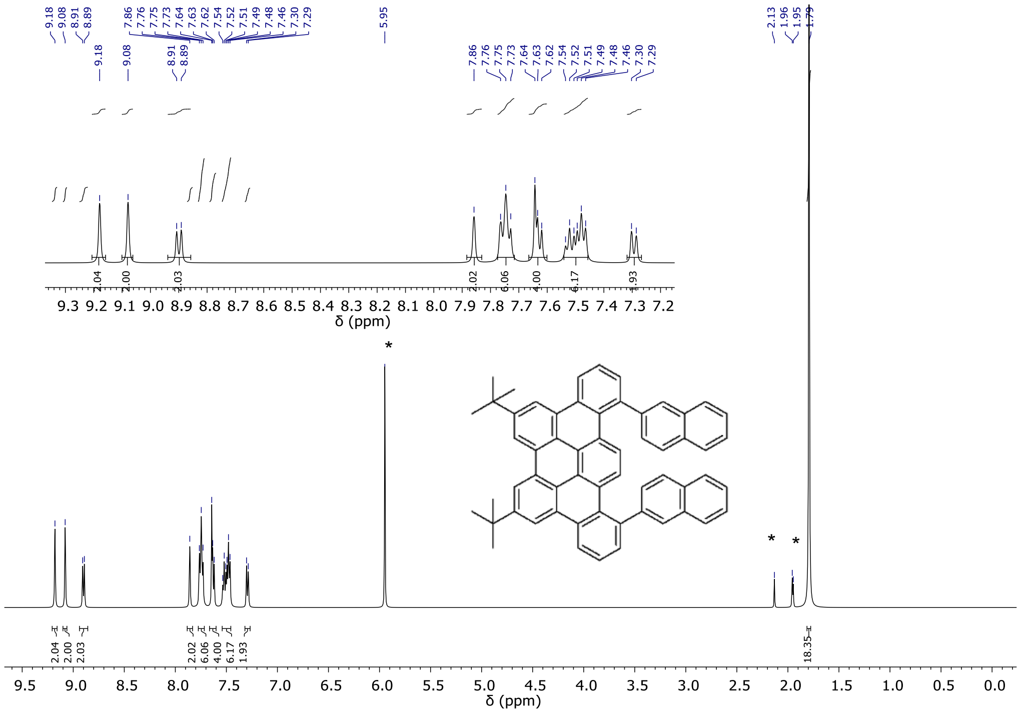

Figure S3. ^{1}H NMR spectrum of **3** in $C_2D_2Cl_4$ (500 MHz, 403 K). The solvent peaks were marked with asterisks.

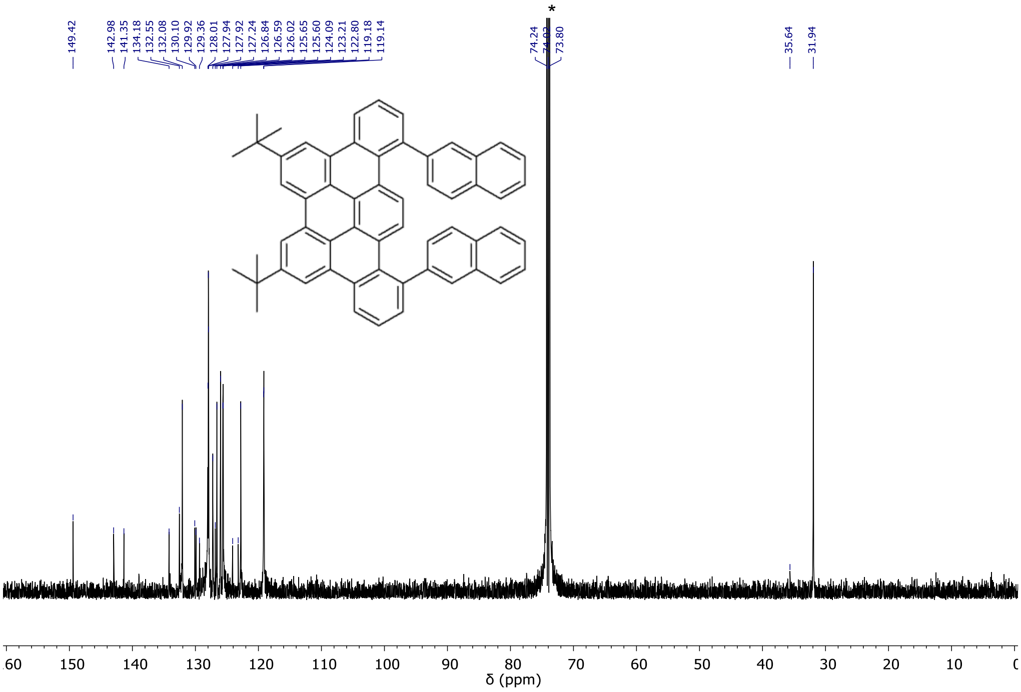

Figure S4. ^{13}C NMR spectrum of **3** in $C_2D_2Cl_4$ (125 MHz, 403 K). The solvent peaks were marked with asterisks.

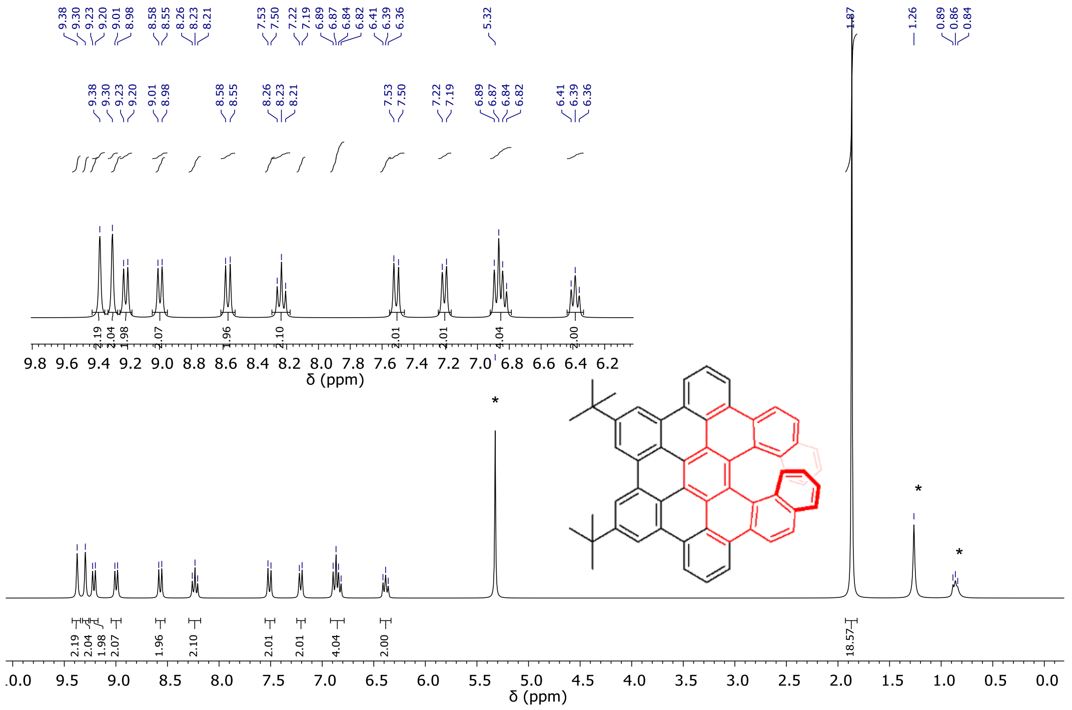

Figure S5. ^{1}H NMR spectrum of **4** in CD_2Cl_2 (300 MHz). The solvent peaks were marked with asterisks.

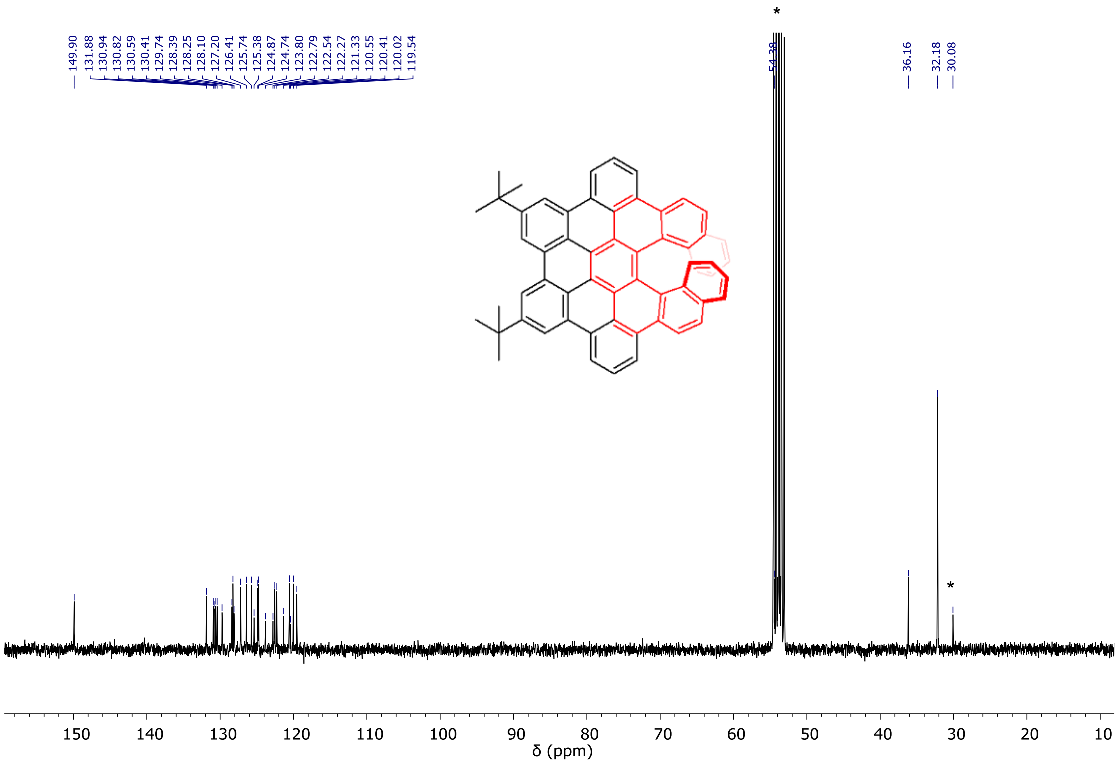

Figure S6. ^{13}C NMR spectrum of **4** in CD_2Cl_2 (75 MHz). The solvent peaks were marked with asterisks.

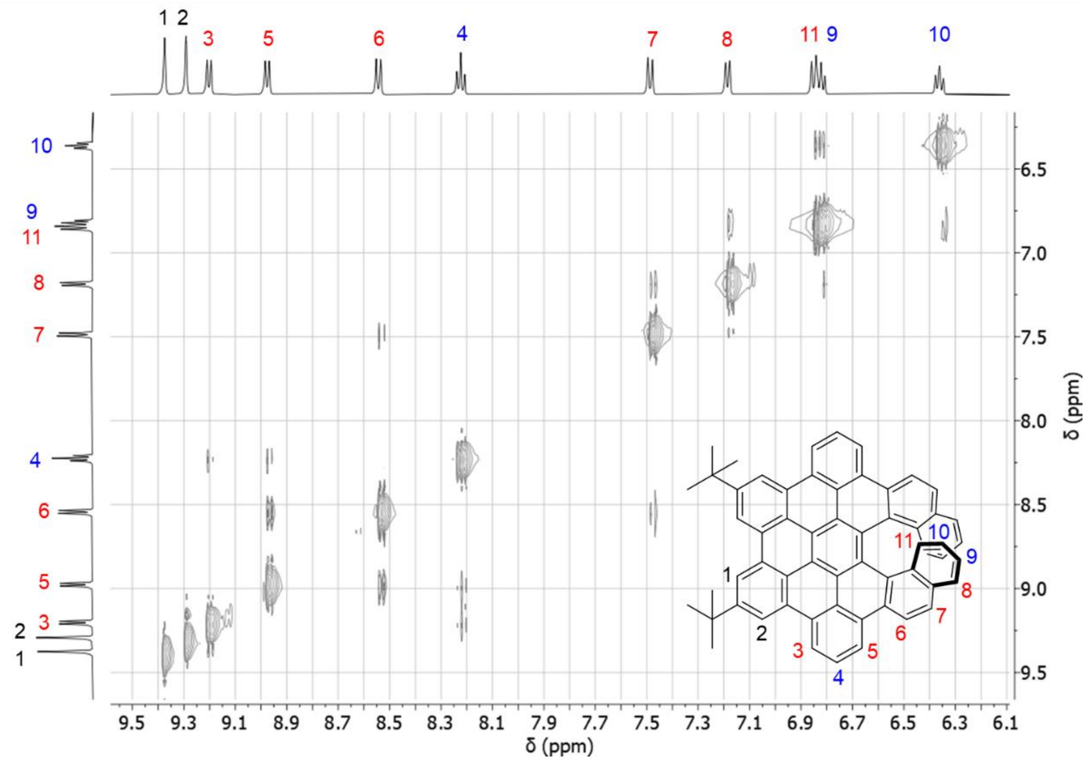

Figure S7. ^{1}H-^{1}H COSY spectrum of **4** in CD_2Cl_2 (300 MHz). Singlet, doublet, and triplet peaks are labeled in black, red, and blue colors, respectively.

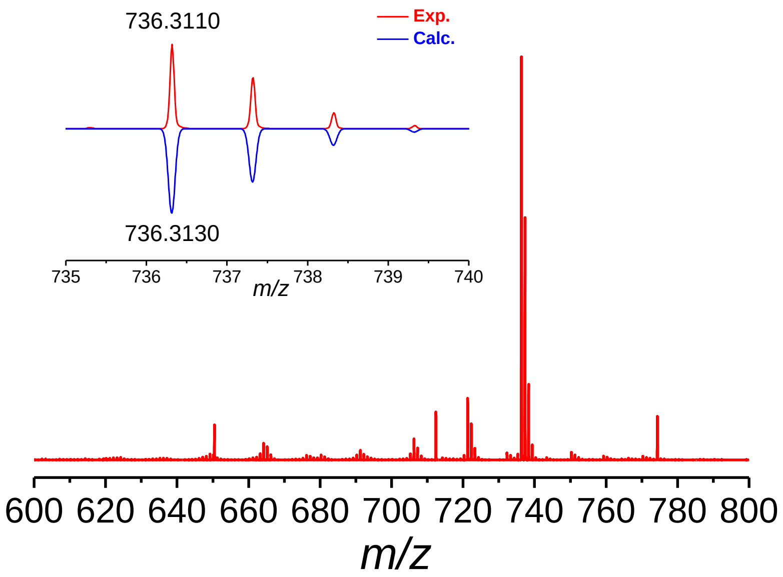

Figure S8. HR MALDI-TOF mass spectrum and isotopic distribution pattern of **4**.

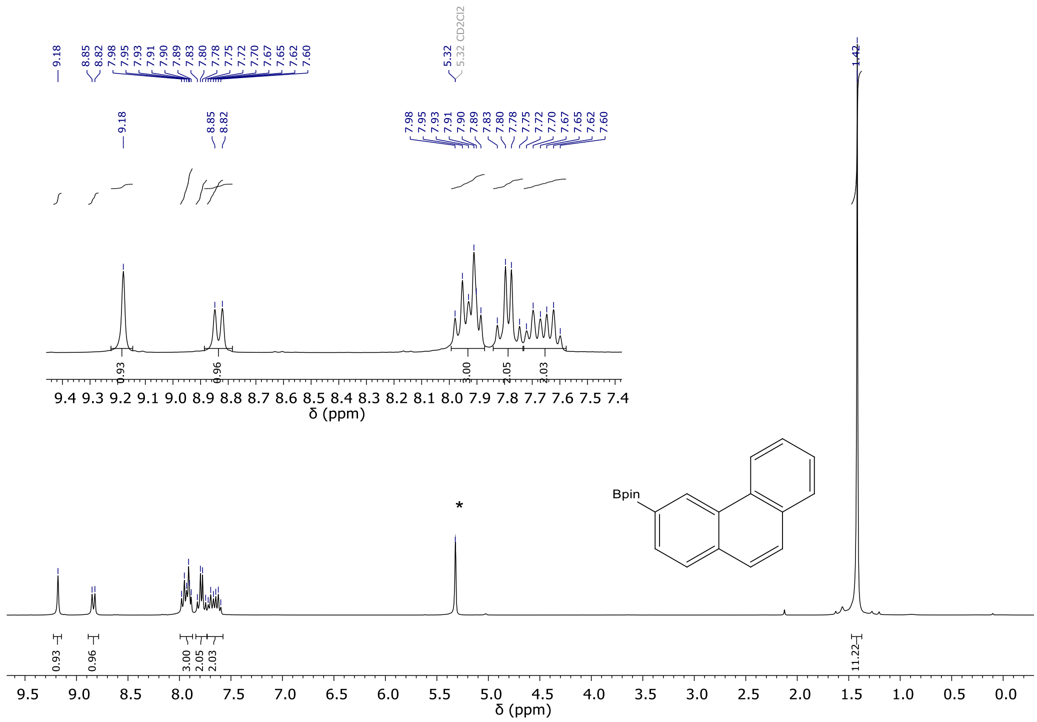

Figure S9. ^{1}H NMR spectrum of **S1** in CD_2Cl_2 (300 MHz). The solvent peaks were marked with asterisks.

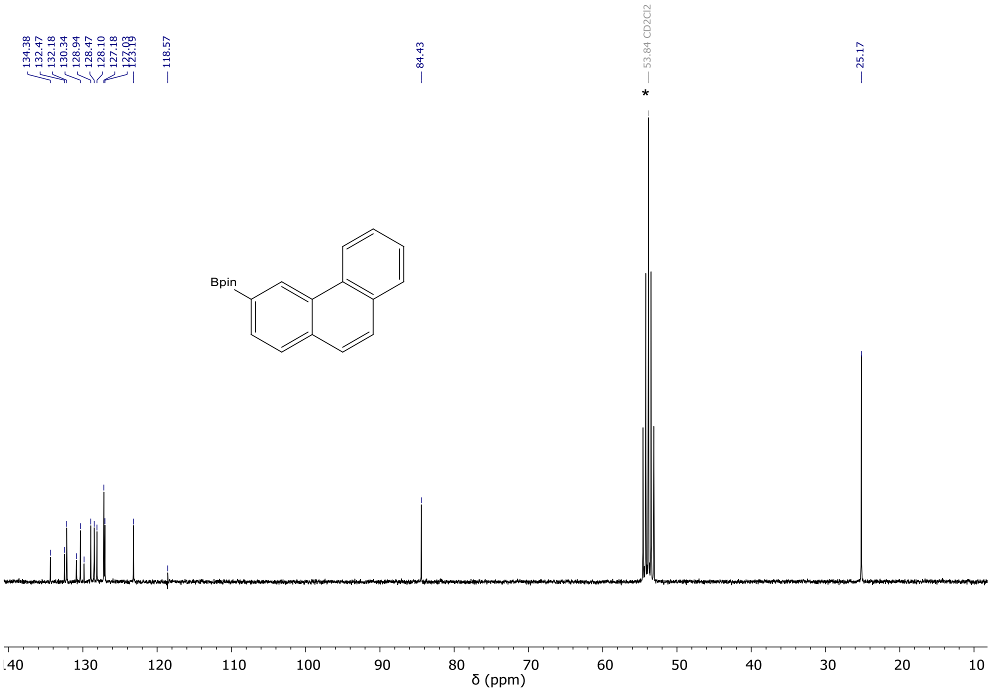

Figure S10. ^{13}C NMR spectrum of **S1** in CD_2Cl_2 (75 MHz). The solvent peaks were marked with asterisks.

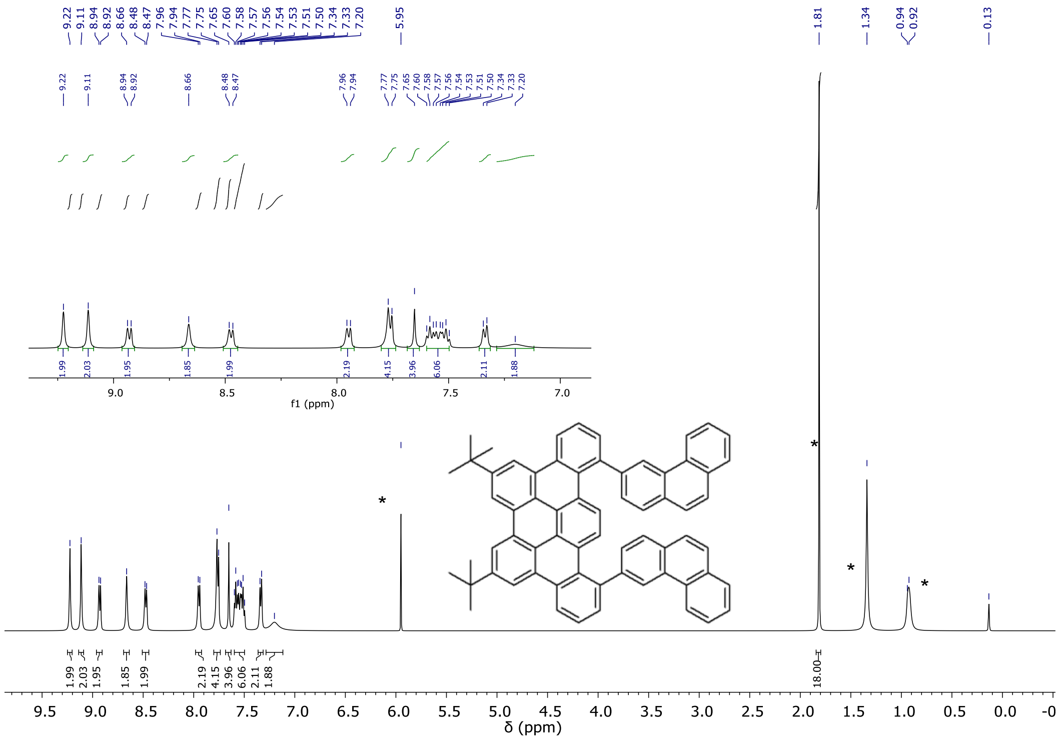

Figure S11. ^{1}H NMR spectrum of **5** in $C_2D_2Cl_4$ (500 MHz, 403 K). The solvent peaks were marked with asterisks.

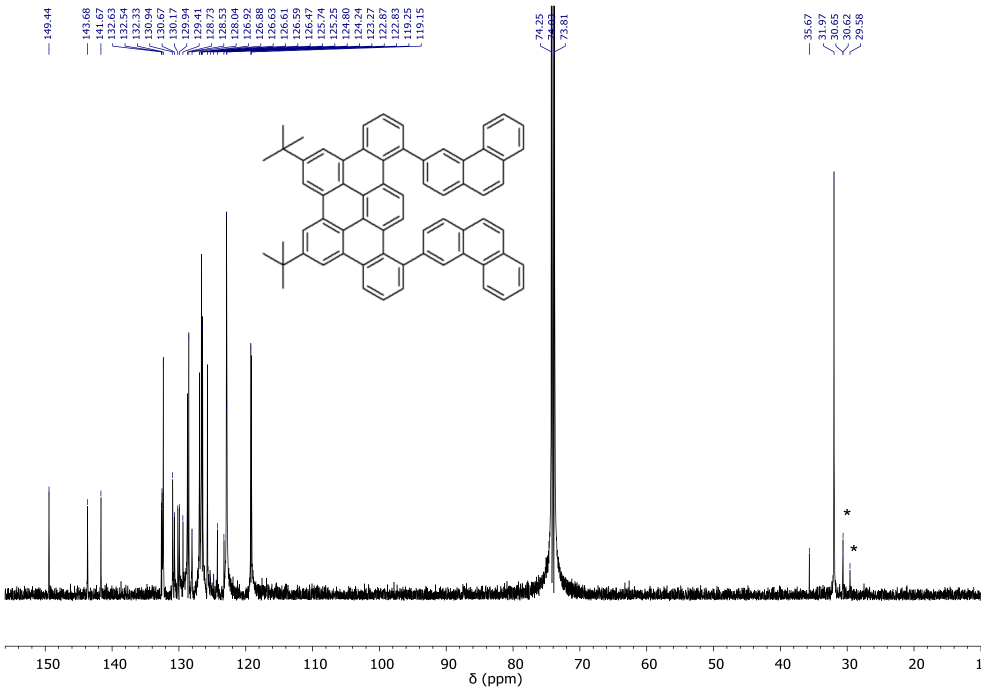

Figure S12. ^{13}C NMR spectrum of **5** in $C_2D_2Cl_4$ (126 MHz, 403 K). The solvent peaks were marked with asterisks.

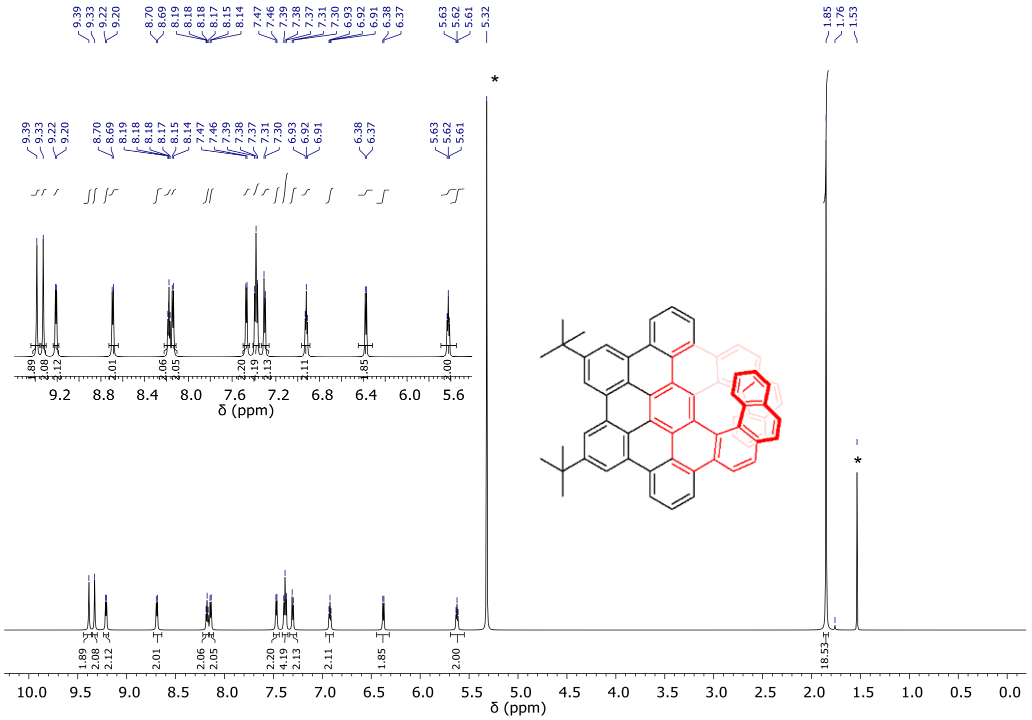

Figure S13. ^{1}H NMR spectrum of **6** in CD_2Cl_2 (700 MHz). The solvent peaks were marked with asterisks.

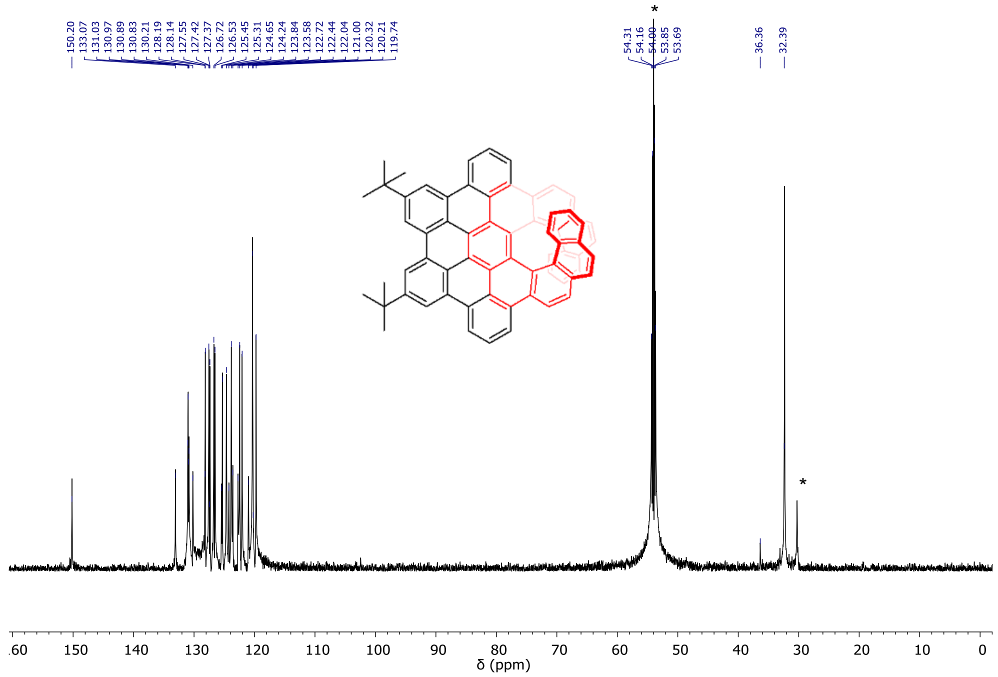

Figure S14. ^{13}C NMR spectrum of **6** in CD_2Cl_2 (176 MHz). The solvent peaks were marked with asterisks.

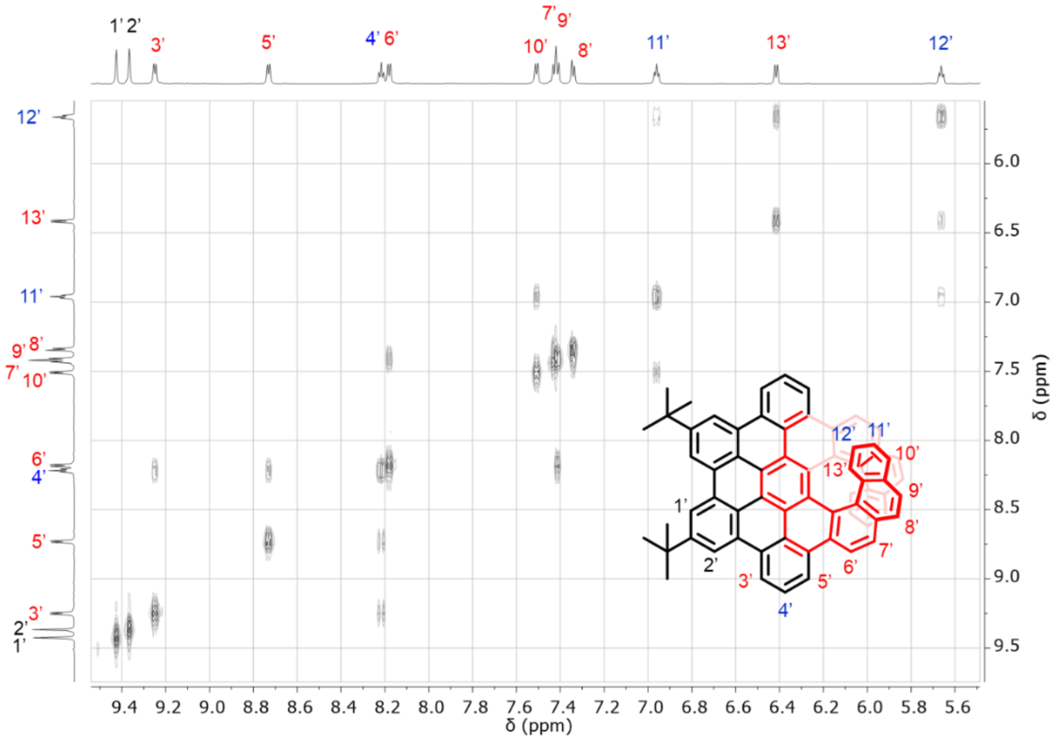

Figure S15. ^{1}H-^{1}H COSY spectrum of **6** in CD_2Cl_2 (700 MHz). Singlet, doublet, and triplet peaks are labeled in black, red, and blue colors, respectively.

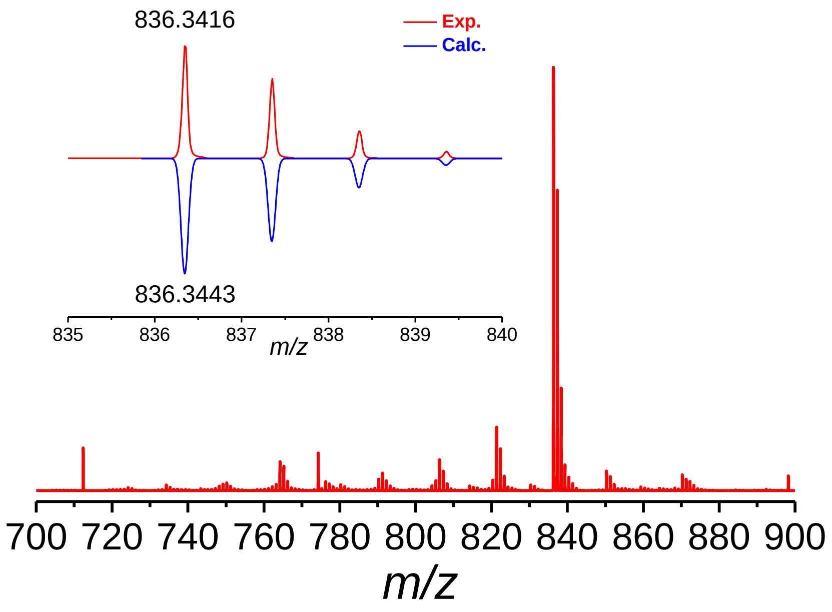

Figure S16. HR MALDI-TOF mass spectrum and isotopic distribution pattern of **6**.

3. X-Ray Single Crystallography

The single crystals of compound **3**, **4**, and **6** suitable for X-ray analysis were obtained by diffusing methanol vapor into the chloroform solution of each compound. The structures were deposited at The Cambridge Crystallographic Data Centre and the data can be obtained free of charge via www.ccdc.cam.ac.uk/structures.

Crystal data for 3 (CCDC number: 2047540)

formula	$C_{58}H_{44}$, $CHCl_3$, 0.25(H_2O)		
molecular weight	864.80 $gmol^{-1}$		
absorption	μ = 2.136 mm^{-1} correction with 6 crystal faces		
transmission	T_{min} = 0.522, T_{max} = 0.959		
crystal size	0.02 x 0.03 x 0.35 mm^3 colourless needle		
space group	P 2_1/c (monoclinic)		
lattice parameters	a = 14.8035(7)Å		
(calculate from	b = 10.8456(5)Å	ß = 98.723(3)	
35193 reflections with	c = 28.4698(13)Å		
3.0° < θ < 67.9°)	V = 4518.0(4)$Å^3$	z = 4	F(000) = 1810
temperature	120K		
density	d_{xray} = 1.271 gcm^{-3}		

<u>data collection</u>

diffractometer	STOE IPDS 2T
radiation	Cu-K_α IµS mirror system
Scan – type	ω scans
Scan – width	1°
scan range	3.0° ≤ θ < 68.7°
	-16 ≤ h ≤ 16 -12 ≤ k ≤ 12 -31 ≤ l ≤ 31
number of reflections:	
measured	39120
unique	7933 (R_{int} = 0.086)
observed	4393 (|F|/σ(F) > 4.0)

<u>data correction, structure solution and refinement</u>

corrections	Lorentz and polarisation correction.
Structure solution	Program: SHELT-2014
refinement	Program: SHELXL-2018 (full matrix). 606 refined parameters, weighting scheme: $w=1/[\sigma^2(F_o^2) + (0.1868*P)^2+16.67*P]$ with $(Max(F_o^2,0)+2*F_c^2)/3$. H-atoms at calculated positions and refined with isotropic displacement parameters, non H- atoms refined anisotropically.
R-values	wR2 = 0.3937 (R1 = 0.1262 for observed reflections, 0.1985 for all reflections)
goodness of fit	S = 1.02
maximum deviation	

of parameters 0.001 * e.s.d
maximum peak height in
diff. Fourier synthesis 1.24, -0.64 eÅ^{-3}
remark structure contains solvent and traces of water

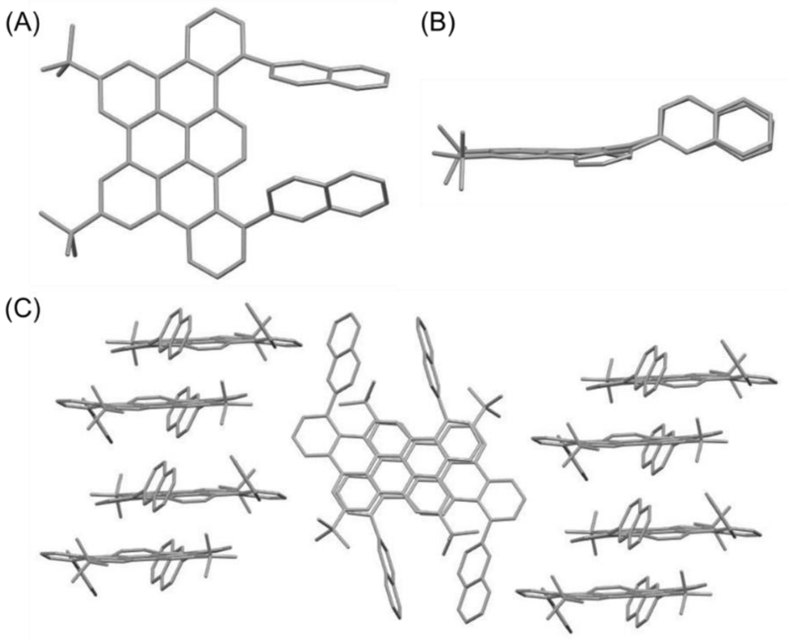

Figure S17. Single-crystal structure of **3.** (A) Top view, (B) side view, and (C) molecular packing. All the hydrogen atoms are removed for clarity.

<u>Crystal data for 4 (CCDC number: 2047541)</u>

formula	2($C_{58}H_{40}$), $CHCl_3$
molecular weight	1593.16 gmol^{-1}
absorption	μ = 0.172 mm^{-1}
crystal size	0.06 x 0.13 x 0.18 mm^3 yellow plate
space group	P 2_1/c (monoclinic)
lattice parameters	a = 26.3941(11)Å
(calculate from	b = 11.3731(6)Å ß = 94.105(3)
12028 reflections with	c = 26.5896(11)Å
2.45° < θ < 26.8°)	V = 7961.3(6)Å^3 z = 4 F(000) = 3336
temperature	120K
density	d_{xray} = 1.329 gcm^{-3}

<u>data collection</u>

diffractometer	STOE IPDS 2T
radiation	Mo-K_α Graphitmonochromator
Scan – type	ω scans
Scan – width	1°
scan range	2° ≤ θ < 28°
	-31 ≤ h ≤ 34 -15 ≤ k ≤ 15 -31 ≤ l ≤ 31
number of reflections:	

measured	40666
unique	18898 (R_{int} = 0.1316)
observed	6989 (|F|/σ(F) > 4.0)

<u>data correction, structure solution and refinement</u>

corrections	Lorentz and polarisation correction.
Structure solution	Program: SHELXT 2014 (Dual Space)
refinement	Program: SHELXL-2018 (full matrix). 1149 refined parameters, weighting scheme: $w=1/[\sigma^2(F_o^2) + (0.1458*P)^2]$ with $(Max(F_o^2,0)+2*F_c^2)/3$. H-atoms at calculated positions and refined with isotropic displacement parameters, non H- atoms refined anisotropically.
R-values	wR2 = 0.3259 (R1 = 0.1030 for observed reflections, 0.2505 for all reflections)
goodness of fit	S = 1.011
maximum deviation of parameters	0.001 * e.s.d
maximum peak height in diff. Fourier synthesis	0.54, -0.48 eÅ^{-3}
remark	*t*-butyl groups are disordered

<u>Crystal data for 6 (CCDC number: 2047542)</u>

moiety formula	$C_{66}H_{44}$	
Formula weight	837.01	
Temperature	120(2) K	
Wavelength, radiation type	0.71073Å, MoKa	
Diffractometer	STOE IPDS 2T	
Crystal system	Monoclinic	
Space group name, number	P 2/c, (13)	
Unit cell dimensions	a = 16.8607(11) Å	a = 90°
	b = 16.1378(11) Å	b = 95.331(5)°
	c = 15.3067(10) Å	g = 90°
Volume	4146.9(5) Å^3	
Number of reflections and range used for lattice parameters	4140<br>2.52° <=θ<= 28.26°	
Z	4	
Density (calculated)	1.341 mg/m^3	
Absorption coefficient	0.076 mm^{-1}	
Absorption correction	None	
F(000)	1760	
Crystal size, colour and form	0.400 x 0.150 x 0.040 mm^3, orange plate	
Theta range for data collection	2.524 to 28.032°.	
Index ranges	-22<=h<=22, -20<=k<=21, -20<=l<=19	
Number of reflections:		
collected	21448	
independent	9871 [R(int) = 0.0646]	
observed [I>2sigma(I)]	5259	
Completeness to theta = 25.2°	99.7 %	
Refinement method	Full-matrix least-squares on F^2	

Data / restraints / parameters	9871 / 36 / 632
Goodness-of-fit on F^2	1.134
Final R indices [I>2sigma(I)]	R1 = 0.0962, wR2 = 0.1831
R indices (all data)	R1 = 0.1887, wR2 = 0.2348
Largest diff. peak and hole	0.332 and -0.293 eÅ^{-3}
remark	structure contains two independent
molecules	both with C_2 symmetry

4. Photophysical Properties and Chiral HPLC analysis

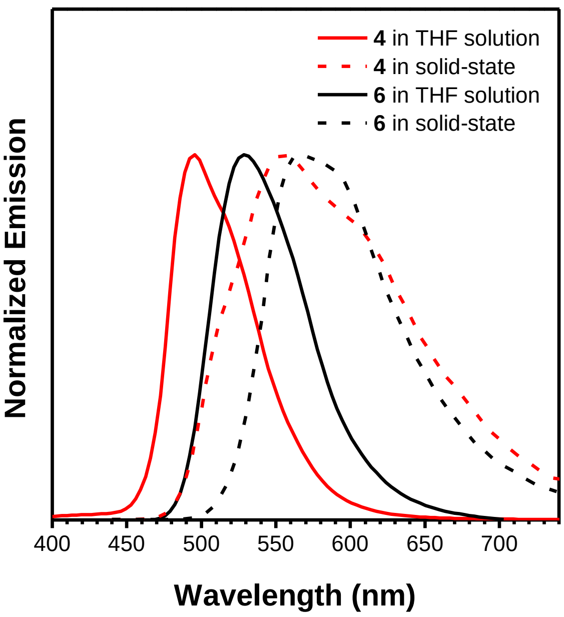

Figure S18. Emission spectra of **4** and **6** in THF solution (10^{-5} M) and solid-state.

Scheme S1. Chemical structures and solution quantum yields of (A-B) π-extended helicenes and (C-D) their pristine counterparts.[2-3]

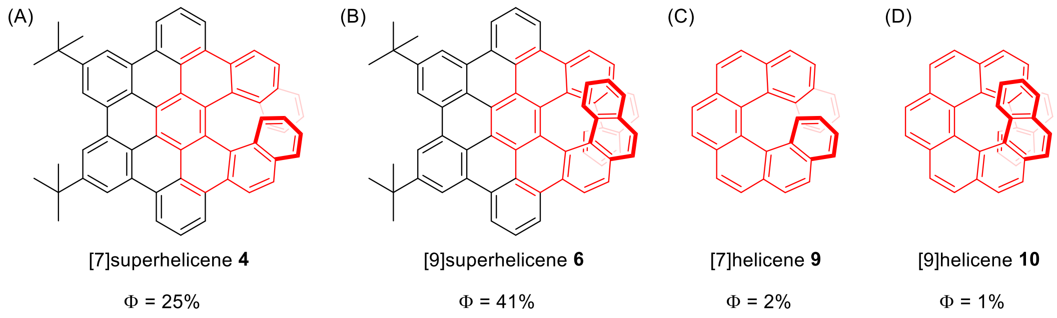

The experimental g_{abs} values of **9** and **10** are estimated from the spectra in the literature to be ~0.017 at 350 nm and ~0.025 at 400 nm, respectively.[4,5] Therefore, there is only a small increase of the experimental g_{abs} values upon going from **9** to **10**.

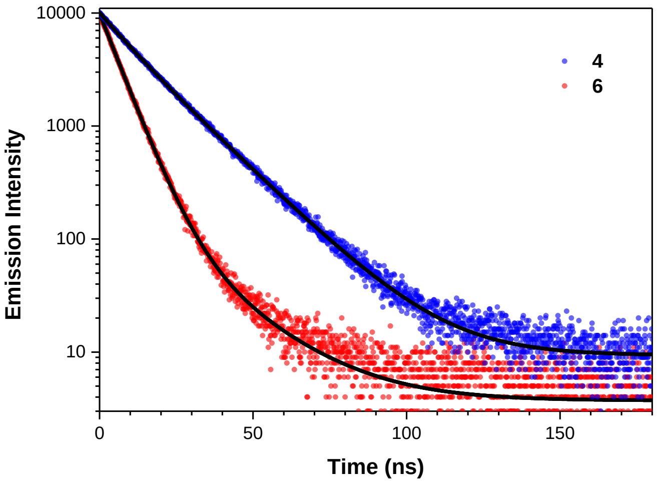

Figure S19. Emission lifetime decay curves for **4** and **6** measured at 624 nm in THF solution and its monoexponential fit. Concentration: 10^{-5} M.

Table S1. Summary of experimental and calculated photophysical properties of **4** and **6**.

	Φ_l[a]	Φ_s[b]	τ_1[c] (ns)	τ_2[c] (ns)	k_r (× 10^7 s^{-1})	k_{nr} (× 10^7 s^{-1})	HOMO (eV)[d]	LUMO (eV)[d]
**4**	0.25	0.17	8.25 (11.5%)	16.71 (88.5%)	1.5	4.6	-5.21	-5.13
**6**	0.41	0.34	6.11 (92.8%)	19.64 (7.22%)	4.5	6.8	-2.06	-2.06

[a] Quantum yield measured in THF solution. Concentration: 10^{-5} M. [b] Quantum yield measured in the solid-state with a calibrated integrating sphere system. [c] Life-time measured in THF solution fitted by a biexponential decay model. Concentration: 10^{-5} M. [d] Calculated by DFT at the B3LYP/6-311G (d,p) level.

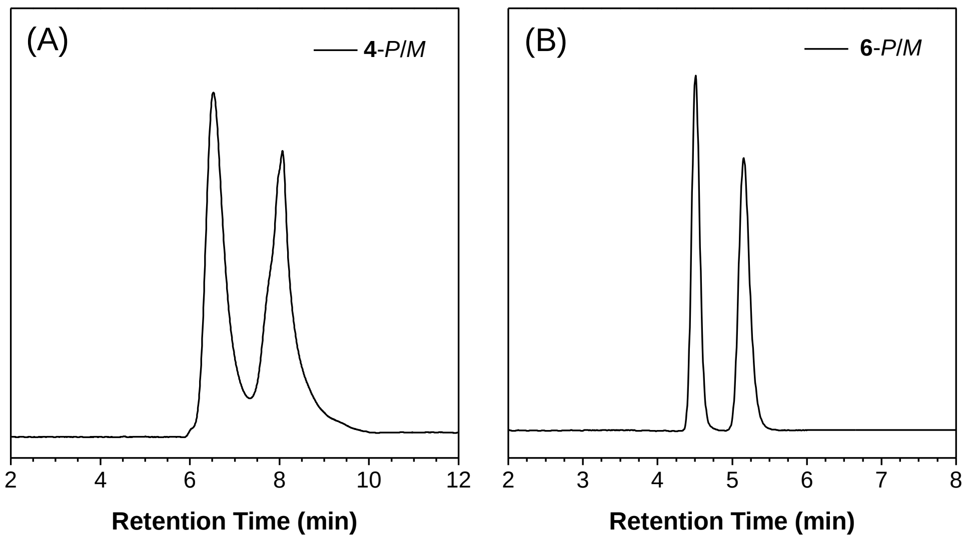

Figure S20. Chiral HPLC analysis of (A) **4** and (B) **6** were performed using Daicel Chiralpak IE HPLC column. Elution for **4**: *n*-heptane/tetrahydrofuran/isopropanol (95/4.3/0.7). Elution for **6**: *n*-hexane/tetrahydrofuran (80/20).

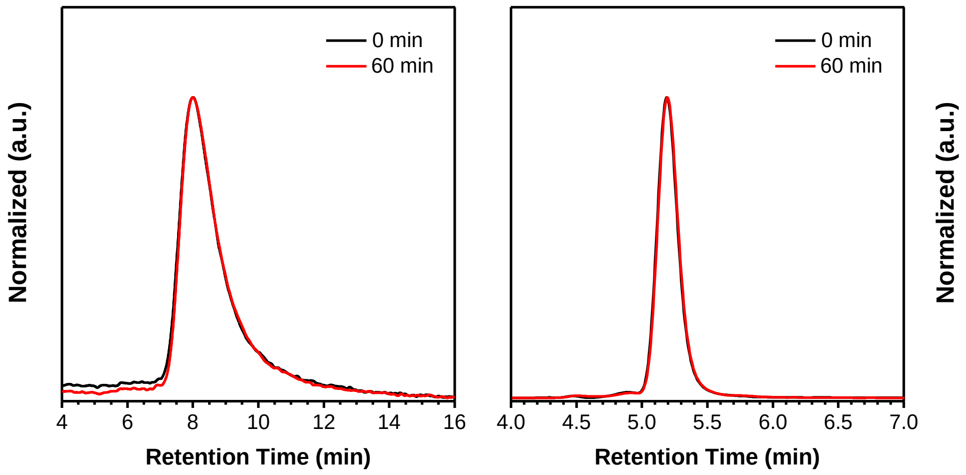

Figure S21. Thermal stability tests of (A) **4**-*M* and (B) **6**-*M* heated in mesitylene at 150 °C for 60 min. The chiral HPLC analyses were performed using Daicel Chiralpak IE HPLC column. Elution for **4**: *n*-heptane/tetrahydrofuran/isopropanol (95/4.3/0.7). Elution for **6**: *n*-hexane/tetrahydrofuran (80/20).

5. DFT Calculations

DFT calculations were performed using the Gaussian 09 software package.[6] Two simple helicenes ([7]helicene **9** and [9]helicene **10**) were optimized at the same level of theory for comparison (Scheme S1). The geometries, molecular orbitals, and MO energies were calculated at the B3LYP/6-311G(d,p) level. The UV-vis absorption and CD spectra were simulated by time-dependent DFT (TD-DFT) calculations at the same level of theory with THF as solvent. The electric and magnetic transition dipole moments of **4** and **6** were calculated by Multiwfn.[7]

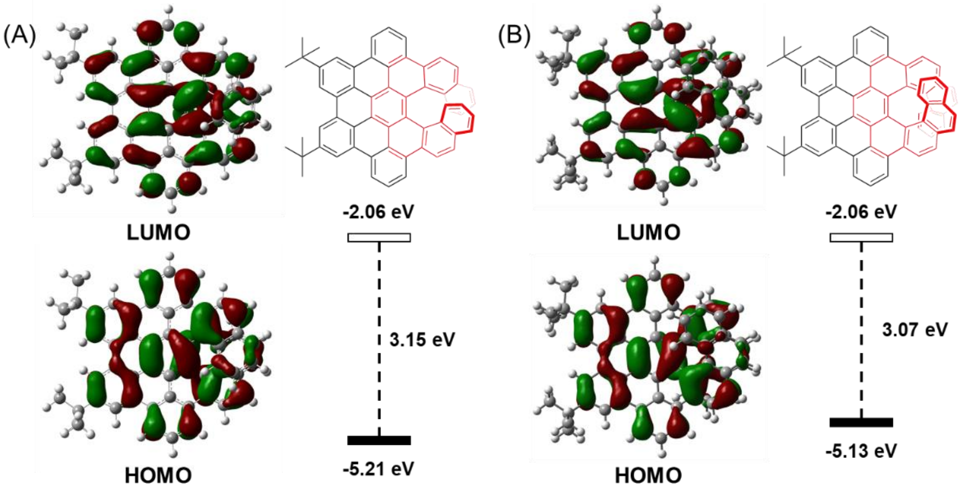

Figure S22. Frontier molecular orbitals of (A) **4** and (B) **6**, and their energies calculated at the B3LYP/6-311G (d,p) level, respectively.

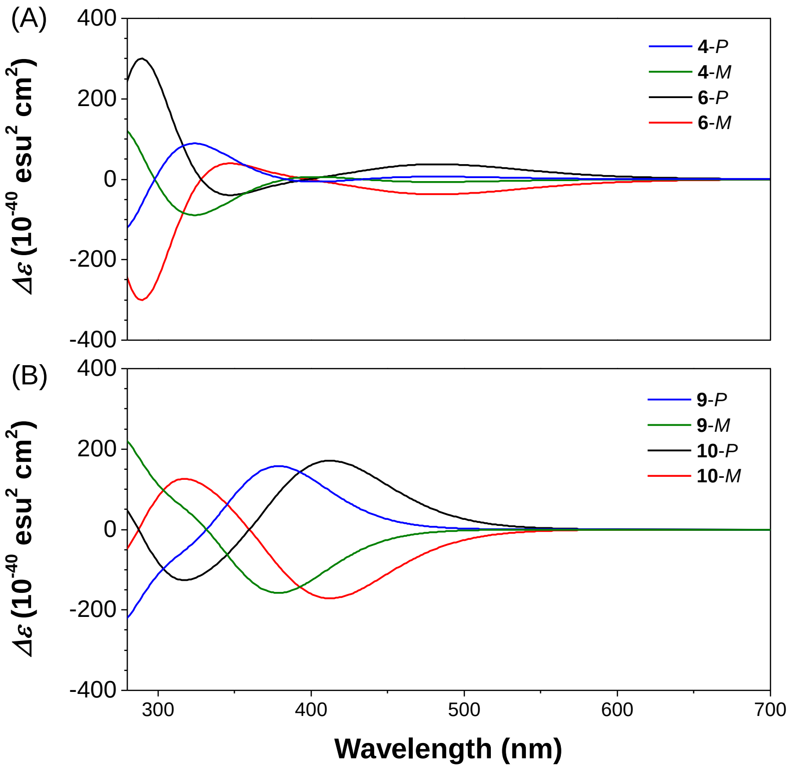

Figure S23. The simulated ECD spectra of (A) **4**-*P*/*M* and **6**-*P*/*M* and (B) **9**-*P*/*M* and **10**-*P*/*M* calculated by TD-DFT at the B3LYP/6-311G(d,p) level.

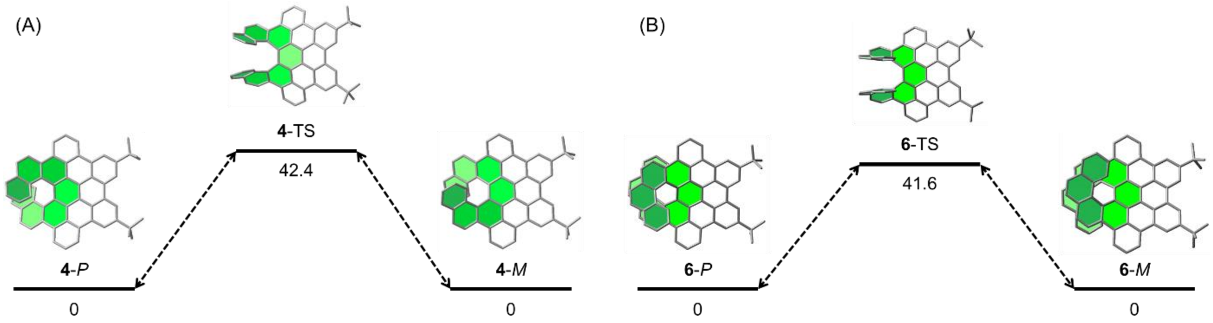

Figure S24. *P*/*M* isomerization barriers of (A) **4** and (B) **6**. The relative Gibbs free energies (unit: kcal mol^{-1}) were calculated at the B3LYP/6-311G(d,p) level. All hydrogen atoms were omitted for clarity.

Table S2. Major transitions of **4** calculated by TD-DFT.

excited state	energy (eV)	wavelength (nm)	oscillator strength (*f*)	Description
1	2.6828	462.14	0.2170	H-1 -> L+1 0.13058
				H -> L 0.68674
2	2.7551	450.02	0.0087	H-2 -> L -0.12708
				H-1 -> L 0.51010
				H -> L+1 -0.44216
				H -> L+2 -0.13812
3	3.0194	410.63	0.0311	H-2 -> L 0.40812
				H-1 -> L 0.23120
				H -> L+2 0.52049
4	3.1079	398.93	0.5956	H-2 -> L 0.10285
				H-1 -> L 0.39637
				H -> L+1 0.50932
				H -> L+2 -0.24375
5	3.2774	378.3	0.1669	H-2 -> L 0.46388
				H-1 -> L -0.11282
				H-1 -> L+1 0.21656
				H-1 -> L+2 0.20820
				H -> L+1 -0.17106
				H -> L+2 -0.31218
6	3.2855	377.36	0.2075	H-4 -> L -0.16061
				H-2 -> L -0.25375
				H-2 -> L+1 0.12661
				H-1 -> L+1 0.40264
				H-1 -> L+2 0.38949
				H -> L+2 0.16700
7	3.3157	373.93	0.0762	H-2 -> L+1 -0.19274
				H-1 -> L+1 0.47164
				H-1 -> L+2 -0.45239
8	3.3942	365.28	0.0644	H-3 -> L 0.34364
				H -> L+3 0.60191
9	3.4671	357.6	0.0539	H-4 -> L -0.11089
				H-2 -> L+1 0.47867
				H-2 -> L+2 0.34234
				H-1 -> L+2 -0.22428
				H -> L+4 0.26967
10	3.5016	354.08	0.0103	H-4 -> L 0.45667
				H-2 -> L+1 0.26895
				H-2 -> L+2 0.12640
				H-1 -> L+1 0.11521
				H -> L+4 -0.40130
11	3.5751	346.8	0.1766	H-3 -> L 0.58563
				H -> L+2 -0.11414
				H -> L+3 -0.32756
12	3.5939	344.98	0.0148	H-5 -> L 0.10336
				H-4 -> L -0.33367
				H-2 -> L+1 -0.18939

				H-2 -> L+2	0.47535
				H -> L+4	-0.31579
13	3.6388	340.72	0.2435	H-4 -> L	-0.26738
				H-2 -> L+1	0.24480
				H-2 -> L+2	-0.26587
				H-1 -> L+2	-0.12587
				H-1 -> L+3	0.38608
				H -> L+4	-0.29206
14	3.7345	332	0.1883	H-4 -> L	0.19392
				H-3 -> L+1	0.31293
				H-2 -> L+1	-0.17396
				H-2 -> L+2	0.18974
				H-2 -> L+3	-0.13152
				H-1 -> L+3	0.46519
				H -> L+4	0.15697
15	3.8292	323.78	0.0065	H-5 -> L	0.60078
				H-3 -> L+1	0.21539
				H-1 -> L+3	-0.13511
				H -> L+4	0.15429
				H -> L+5	0.16106
16	3.8329	323.48	0.0153	H-4 -> L+1	-0.25021
				H-1 -> L+4	0.62554
17	3.8642	320.85	0.0382	H-5 -> L	-0.11961
				H-3 -> L+1	0.22901
				H-3 -> L+2	0.36246
				H-2 -> L+3	-0.32713
				H-1 -> L+3	-0.22366
				H -> L+5	-0.32157
18	3.8825	319.34	0.0105	H-4 -> L+1	0.30013
				H-4 -> L+2	0.48832
				H-2 -> L+4	0.31495
				H -> L+6	-0.10785
19	3.9261	315.8	0.0494	H-5 -> L	-0.18246
				H-3 -> L+2	0.14340
				H-2 -> L+3	-0.32444
				H -> L+5	0.54675
20	3.937	314.92	0.0277	H-6 -> L	-0.22603
				H-5 -> L+1	-0.11487
				H-4 -> L+1	0.43428
				H-2 -> L+4	-0.12268
				H-1 -> L+4	0.17292
				H-1 -> L+5	-0.16418
				H -> L+6	0.36350

Table S3. Major transitions of **6** calculated by TD-DFT.

excited state	energy (eV)	wavelength (nm)	oscillator strength (*f*)	Description	
1	2.5788	480.78	0.1458	H -> L	0.68784
2	2.7237	455.2	0.0002	H-2 -> L	0.14540
				H-1 -> L	0.47390
				H -> L+1	-0.47339
				H -> L+2	0.14781
3	2.9613	418.69	0.0019	H-2 -> L	0.46347
				H-1 -> L	-0.16555
				H -> L+1	0.12796
				H -> L+2	0.47424
4	2.9999	413.3	0.0007	H-3 -> L	0.56818
				H -> L+3	-0.39299
5	3.0838	402.05	0.6397	H-2 -> L	-0.12261
				H-1 -> L	0.46364
				H -> L+1	0.47577
				H -> L+2	0.15822
6	3.1262	396.6	0.0535	H-3 -> L	0.35902
				H-2 -> L+1	-0.10589
				H-1 -> L+1	-0.18137
				H-1 -> L+2	0.10932
				H -> L+3	0.54304
7	3.1678	391.39	0.1236	H-2 -> L	0.46841
				H-1 -> L	0.14016
				H -> L+1	0.14261
				H -> L+2	-0.45010
8	3.2919	376.63	0.2289	H-4 -> L	-0.18564
				H-3 -> L	0.15989
				H-2 -> L+2	-0.22052
				H-1 -> L+1	0.55468
				H-1 -> L+2	-0.18437
				H -> L+3	0.12885
9	3.3316	372.14	0.0058	H-2 -> L+1	0.44710
				H-1 -> L+1	0.11991
				H-1 -> L+2	0.50335
10	3.3369	371.56	0.0142	H-5 -> L	-0.14854
				H-3 -> L+2	-0.10278
				H -> L+4	0.66598
11	3.4142	363.14	0.0085	H-4 -> L	0.18773
				H-2 -> L+1	0.38156
				H-2 -> L+2	-0.36129
				H-1 -> L+1	-0.22708

				H-1 -> L+2	-0.33973
12	3.437	360.73	0.0035	H-5 -> L	0.22207
				H-4 -> L	-0.11444
				H-3 -> L+1	0.52102
				H-3 -> L+2	-0.22991
				H-1 -> L+3	-0.30793
13	3.4648	357.84	0.0045	H-5 -> L	0.42705
				H-4 -> L	-0.30380
				H-3 -> L+2	-0.11023
				H-2 -> L+3	0.18288
				H-1 -> L+3	0.38348
14	3.4925	355.01	0.0513	H-5 -> L	0.32883
				H-4 -> L	0.49612
				H-3 -> L+3	0.15211
				H-2 -> L+1	-0.19803
				H-2 -> L+2	-0.13590
				H-1 -> L+1	0.18201
15	3.5248	351.75	0.0107	H-5 -> L	0.19279
				H-3 -> L+1	-0.13457
				H-3 -> L+2	0.37604
				H-3 -> L+4	0.16981
				H-2 -> L	-0.10705
				H-2 -> L+3	0.33083
				H-1 -> L+3	-0.33589
				H -> L+4	0.13707
16	3.5583	348.44	0.0014	H-5 -> L	-0.16693
				H-3 -> L+1	0.41082
				H-3 -> L+2	0.32189
				H-2 -> L+3	0.19821
				H-1 -> L+3	0.33138
17	3.6073	343.7	0.19	H-4 -> L	0.15031
				H-2 -> L+1	0.25561
				H-2 -> L+2	0.45402
				H-1 -> L+2	-0.18520
				H-1 -> L+4	-0.12550
				H -> L+5	0.34268
18	3.6465	340.01	0.117	H-3 -> L+3	0.47617
				H-2 -> L+1	0.14314
				H-2 -> L+2	0.23971
				H-1 -> L+2	-0.10480
				H -> L+5	-0.39923
19	3.6734	337.52	0.3253	H-5 -> L	-0.18991
				H-3 -> L+2	-0.37765
				H-2 -> L+3	0.48409

excited state	energy (eV)	wavelength (nm)	oscillator strength (f)	Description
				H -> L+4 -0.11703
				H -> L+6 0.10566
20	3.7123	333.98	0.0578	H-6 -> L -0.14316
				H-5 -> L+1 -0.12439
				H-5 -> L+2 0.12595
				H-4 -> L+2 -0.10293
				H-3 -> L+3 0.23774
				H-2 -> L+4 0.13122
				H-1 -> L+4 0.43716
				H -> L+5 0.30858
				H -> L+6 -0.16519

Table S4. Major transitions of **9** calculated by TD-DFT.

excited state	energy (eV)	wavelength (nm)	oscillator strength (f)	Description
1	3.0775	402.87	0.0003	H-1 -> L+1 0.43496<br>H -> L 0.54981
2	3.2486	381.65	0.0175	H-1 -> L 0.63146<br>H -> L+1 -0.31178
3	3.3184	373.63	0.1583	H-2 -> L 0.22515<br>H-1 -> L 0.30019<br>H -> L+1 0.59583
4	3.5217	352.06	0.0661	H-3 -> L+1 -0.15653<br>H-2 -> L 0.60561<br>H-1 -> L+2 -0.25003<br>H -> L+1 -0.18623
5	3.573	347	0.0457	H-2 -> L+1 0.14909<br>H-1 -> L+1 0.52747<br>H -> L -0.42394
6	3.6494	339.74	0.0486	H-3 -> L 0.15559<br>H-2 -> L+1 0.63913<br>H -> L+2 0.21332
7	3.8715	320.25	0.0128	H-3 -> L -0.47023<br>H -> L+2 0.50828
8	3.9461	314.2	0.129	H-3 -> L+1 0.17419<br>H-2 -> L 0.2598<br>H-1 -> L+2 0.48462<br>H -> L+3 0.37733
9	4.001	309.88	0.005	H-3 -> L+1 0.56963<br>H-1 -> L+2 -0.3672<br>H -> L+3 0.19155
10	4.0397	306.91	0.1392	H-3 -> L -0.39182<br>H-2 -> L+1 0.16032<br>H-1 -> L+3 0.42877<br>H -> L+2 -0.33438

Table S5. Major transitions of **10** calculated by TD-DFT.

excited state	energy (eV)	wavelength (nm)	oscillator strength (*f*)	Description
1	2.9097	426.1	0.0000	H-1 -> L 0.52293<br>H -> L+1 0.45418
2	3.0199	410.55	0.0578	H -> L 0.69454
3	3.0746	403.26	0.0440	H-2 -> L -0.27179<br>H-1 -> L+1 0.64803
4	3.203	387.08	0.0190	H-3 -> L+1 -0.14322<br>H-2 -> L 0.60734<br>H-1 -> L+1 0.26719<br>H -> L+2 -0.16632
5	3.2785	378.17	0.0411	H-3 -> L 0.11976<br>H-2 -> L+1 0.59136<br>H-1 -> L -0.16428<br>H -> L+1 0.30634
6	3.3311	372.2	0.0129	H-3 -> L 0.11863<br>H-2 -> L+1 0.31748<br>H-1 -> L 0.43436<br>H -> L+1 -0.41524
7	3.554	348.85	0.0019	H-3 -> L 0.58008<br>H-2 -> L+1 -0.11676<br>H-1 -> L+2 -0.35418
8	3.5701	347.29	0.0214	H-3 -> L+1 0.48207<br>H-2 -> L 0.18282<br>H-1 -> L+3 0.39203<br>H -> L+2 0.23025
9	3.6336	341.22	0.0094	H-3 -> L+1 -0.40162<br>H-2 -> L+2 -0.10639<br>H-1 -> L+3 0.10162<br>H -> L+2 0.55609
10	3.6616	338.6	0.0447	H-3 -> L 0.27154<br>H-1 -> L+2 0.51336<br>H -> L+3 -0.38152

The Cartesian coordinates of all the optimized structures are listed as follows:

4-*P*

	X	Y	Z		X	Y	Z
C	3.910800	-0.746922	1.458173	C	4.823895	-1.155520	-2.717008
C	4.826727	1.134046	2.721918	C	6.047606	-0.479049	-2.902130
C	3.792656	0.553658	2.016833	C	6.186003	0.806290	-2.428675
C	5.112264	-1.470162	1.740655	C	-7.040701	-2.752128	-0.106613
C	6.173558	-0.83879	2.436656	C	-5.841137	-4.521674	-1.400367
C	6.044399	0.447551	2.91001	C	-5.843549	-4.568349	1.124074
C	3.792796	-0.566565	-2.014644	C	-5.618259	5.180233	0.151788
C	1.676963	-0.683638	0.205861	C	-6.611027	3.287966	-1.143126

C	1.683202	0.688159	-0.207862	C	-6.600767	3.238940	1.381332
C	0.462887	1.410433	-0.126803	H	4.694183	2.125409	3.144892
C	-0.774281	0.718471	-0.003377	H	2.859925	1.091858	1.919534
C	-0.780361	-0.693271	0.000748	H	7.083981	-1.405109	2.615019
C	0.450625	-1.395517	0.124013	H	6.858823	0.919281	3.451803
C	-2.025769	-1.412791	-0.046188	H	2.85542	-1.097065	-1.919735
C	-2.012762	1.448702	0.043971	H	-5.397675	-0.915452	-0.052524
C	-3.261904	-0.711331	-0.03055	H	-3.252776	-4.592872	-0.128491
C	-4.457761	-1.447359	-0.064872	H	-3.209751	4.638585	0.122213
C	-4.480720	-2.840694	-0.10462	H	-5.391074	0.982967	0.061598
C	-3.251354	-3.510065	-0.121721	H	-1.577054	-5.494735	-0.602266
C	-2.027330	-2.835121	-0.104907	H	0.57818	-6.658955	-0.616365
C	-2.003383	2.868181	0.100574	H	2.656447	-5.456306	-0.135213
C	-3.223882	3.558326	0.118247	H	2.699498	5.454208	0.128667
C	-4.453970	2.899650	0.105231	H	0.629843	6.673439	0.604192
C	-4.441100	1.501731	0.069168	H	-1.533840	5.526447	0.590412
C	-3.257002	0.754714	0.032336	H	4.167716	-4.561668	0.706619
C	-5.790547	-3.651899	-0.121637	H	6.110621	-3.387997	1.590470
C	-5.799768	3.651023	0.123346	H	6.141774	3.356034	-1.583129
C	0.462565	-2.836900	0.076266	H	4.206386	4.545656	-0.704389
C	0.486041	2.851055	-0.080356	H	4.684293	-2.145809	-3.140223
C	-0.748928	-3.554278	-0.134978	H	6.859517	-0.957476	-3.441814
C	-0.677159	-4.932124	-0.384972	H	7.101408	1.365200	-2.604878
C	0.544842	-5.595415	-0.399533	H	-7.941396	-3.373822	-0.123514
C	1.722086	-4.908606	-0.130763	H	-7.086277	-2.131739	0.794491
C	1.707297	-3.529848	0.131852	H	-7.079445	-2.093131	-0.980261
C	1.736265	3.534373	-0.135622	H	-6.764220	-5.111642	-1.423833
C	1.761177	4.913329	0.124228	H	-5.000291	-5.219332	-1.452365
C	0.588589	5.609658	0.389857	H	-5.812975	-3.898186	-2.299822
C	-0.638378	4.956090	0.375327	H	-5.004505	-5.269534	1.151079
C	-0.720681	3.577783	0.128574	H	-5.814331	-3.978201	2.045672
C	2.899929	-2.798449	0.555702	H	-6.768152	-5.156467	1.125419
C	2.923767	2.792959	-0.556273	H	-6.598660	5.666838	0.166630
C	4.107198	-3.492779	0.869564	H	-5.073976	5.510171	1.042715
C	5.193134	-2.844220	1.382199	H	-5.083998	5.544278	-0.731870
C	2.847232	-1.401101	0.717685	H	-7.573964	3.810940	-1.141277
C	2.860126	1.395916	-0.717792	H	-6.816230	2.215238	-1.203496
C	3.920139	0.733021	-1.455758	H	-6.069835	3.575319	-2.050362
C	5.128149	1.446372	-1.735457	H	-6.054557	3.495575	2.294774
C	5.219355	2.819805	-1.377077	H	-7.565856	3.757369	1.404825
C	4.137464	3.477287	-0.867374	H	-6.800714	2.163734	1.403998

4-*M*

	X	Y	Z		X	Y	Z
C	-3.905389	-0.742931	1.457196	C	-4.814010	-1.155117	-2.717308
C	-4.814237	1.134074	2.724379	C	-6.037425	-0.481814	-2.899645

C	-3.784553	0.554643	2.017420	C	-6.178335	0.799614	-2.424281
C	-5.105532	-1.463509	1.739173	C	7.032582	-2.749158	-0.099066
C	-6.163804	-0.831605	2.435816	C	5.833481	-4.570422	1.118942
C	-6.031461	0.450782	2.911059	C	5.837305	-4.510986	-1.404464
C	-3.786437	-0.56713	-2.014314	C	5.610389	5.175233	0.156712
C	-1.674341	-0.681928	0.206290	C	6.593954	3.233719	1.381726
C	-1.680557	0.686653	-0.209137	C	6.603258	3.287382	-1.141830
C	-0.462230	1.407897	-0.129863	H	-4.679419	2.122207	3.149439
C	0.773030	0.717152	-0.005255	H	-2.853358	1.092012	1.920066
C	0.778972	-0.692239	0.001264	H	-7.074507	-1.394190	2.613256
C	-0.450179	-1.393006	0.125713	H	-6.843038	0.922755	3.453235
C	2.022842	-1.411291	-0.045597	H	-2.850497	-1.096825	-1.920436
C	2.010015	1.446700	0.042254	H	5.389452	-0.915747	-0.050692
C	3.257074	-0.711255	-0.030114	H	3.246267	-4.586554	-0.133100
C	4.451180	-1.446801	-0.063698	H	3.205545	4.631125	0.124358
C	4.473794	-2.837598	-0.104220	H	5.382506	0.980251	0.062465
C	3.246244	-3.505709	-0.122431	H	1.570887	-5.488819	-0.597559
C	2.024276	-2.831586	-0.104269	H	-0.581638	-6.647711	-0.617594
C	2.000829	2.863866	0.097891	H	-2.65525	-5.446078	-0.13502
C	3.219904	3.552746	0.117628	H	-2.696551	5.444999	0.125034
C	4.447770	2.895142	0.105714	H	-0.630867	6.662969	0.599452
C	4.434938	1.499457	0.069385	H	1.529718	5.520256	0.580087
C	3.252488	0.753687	0.032106	H	-4.164696	-4.551325	0.707878
C	5.783062	-3.648437	-0.121885	H	-6.103542	-3.378465	1.588591
C	5.792714	3.646872	0.125187	H	-6.135553	3.347129	-1.578078
C	-0.462675	-2.832664	0.079149	H	-4.203103	4.535831	-0.704943
C	-0.485566	2.846743	-0.085250	H	-4.672683	-2.142182	-3.142731
C	0.746348	-3.549694	-0.132339	H	-6.847141	-0.960470	-3.438732
C	0.673105	-4.92533	-0.383285	H	-7.094140	1.354843	-2.598590
C	-0.547394	-5.586271	-0.399476	H	7.931221	-3.371297	-0.115778
C	-1.722202	-4.899897	-0.130730	H	7.074217	-2.088152	-0.969185
C	-1.706162	-3.523626	0.133910	H	7.075198	-2.134185	0.804090
C	-1.734333	3.528486	-0.139572	H	6.759506	-5.153599	1.121038
C	-1.759785	4.905239	0.120881	H	4.997935	-5.273611	1.137805
C	-0.589233	5.601012	0.385108	H	5.797444	-3.984852	2.041469
C	0.635935	4.949314	0.369163	H	4.999396	-5.209482	-1.459925
C	0.719040	3.572870	0.122975	H	5.807862	-3.882918	-2.298886
C	-2.896842	-2.792109	0.557819	H	6.761330	-5.096750	-1.429384
C	-2.920438	2.787041	-0.558815	H	6.589964	5.660275	0.170979
C	-4.103617	-3.484333	0.870813	H	5.075870	5.539982	-0.724641
C	-5.186924	-2.836272	1.38186	H	5.068727	5.502341	1.048459
C	-2.843298	-1.397699	0.718333	H	7.555342	3.755889	1.406957
C	-2.856376	1.392875	-0.718552	H	6.797502	2.160707	1.400087
C	-3.915752	0.729451	-1.453769	H	6.045943	3.484946	2.293825
C	-5.122663	1.440213	-1.731713	H	6.059920	3.573317	-2.046447
C	-5.213831	2.812412	-1.374576	H	7.562875	3.813442	-1.140069
C	-4.133900	3.469341	-0.867854	H	6.811073	2.216742	-1.202129

4-TS

	X	Y	Z		X	Y	Z
C	-4.014585	-1.680111	0.634396	C	-4.960090	1.654889	2.887357
C	-4.950843	-1.682434	2.880201	C	-6.199255	2.103992	2.396326
C	-3.904441	-1.42883	2.020704	C	-6.331837	2.405666	1.059384
C	-5.216695	-2.298305	0.169065	C	6.833999	-2.847655	1.210595
C	-6.314102	-2.439567	1.04853	C	5.314143	-4.731879	1.823621
C	-6.185788	-2.140027	2.38635	C	6.101175	-4.450204	-0.558416
C	-3.912994	1.411468	2.025756	C	5.499409	5.213910	0.529034
C	-1.684705	-0.723402	-0.098623	C	6.070534	3.443163	2.193585
C	-1.690829	0.729147	-0.097583	C	6.803425	3.216547	-0.211754
C	-0.447201	1.389598	-0.311077	H	-4.821895	-1.52374	3.944765
C	0.781337	0.711167	-0.126701	H	-2.950422	-1.102698	2.412341
C	0.787133	-0.684800	-0.127248	H	-7.235682	-2.86608	0.666211
C	-0.435547	-1.373186	-0.312136	H	-7.016407	-2.299271	3.064553
C	2.030783	-1.395529	-0.043448	H	-2.955891	1.091786	2.415211
C	2.018473	1.432140	-0.043466	H	5.276551	-0.963560	0.861194
C	3.220822	-0.71286	0.311539	H	3.251081	-4.561602	-0.220496
C	4.374593	-1.472938	0.558005	H	3.213675	4.607009	-0.222886
C	4.400196	-2.859665	0.412673	H	5.271685	1.025895	0.851098
C	3.240883	-3.491634	-0.057376	H	1.893469	-5.107987	-1.746849
C	2.066091	-2.785877	-0.312650	H	-0.082026	-5.983195	-2.914000
C	2.044145	2.819517	-0.312964	H	-2.234816	-4.822301	-2.780164
C	3.217314	3.538994	-0.060862	H	-2.275633	4.830958	-2.767629
C	4.377192	2.915561	0.404406	H	-0.132019	6.008898	-2.899775
C	4.359839	1.524089	0.551126	H	1.851286	5.146318	-1.737343
C	3.216412	0.755269	0.311051	H	-3.986226	-3.780748	-2.609058
C	5.652115	-3.703277	0.719073	H	-6.134607	-3.315256	-1.519471
C	5.667214	3.695386	0.722107	H	-6.162532	3.289223	-1.50680
C	-0.356090	-2.695647	-0.87989	H	-4.019178	3.774656	-2.597587
C	-0.378305	2.713045	-0.875931	H	-4.828368	1.494801	3.95138
C	0.882315	-3.387657	-0.934551	H	-7.030141	2.255132	3.076056
C	0.951942	-4.580335	-1.663171	H	-7.257315	2.825801	0.679417
C	-0.162032	-5.069214	-2.336334	H	7.689657	-3.494329	1.422003
C	-1.377097	-4.400135	-2.273731	H	7.151105	-2.120002	0.458472
C	-1.504366	-3.220589	-1.527522	H	6.592152	-2.309804	2.131296
C	-1.531606	3.231348	-1.520417	H	6.190966	-5.345488	2.052788
C	-1.414221	4.413748	-2.263341	H	4.507308	-5.402996	1.521175
C	-0.204139	5.092392	-2.325053	H	5.001830	-4.228113	2.742360
C	0.914309	4.610546	-1.654726	H	5.325692	-5.122854	-0.931551
C	0.855494	3.414273	-0.930166	H	6.344293	-3.744941	-1.357703
C	-2.795099	-2.619654	-1.214250	H	6.991621	-5.052063	-0.352613
C	-2.817171	2.619471	-1.207230	H	6.433459	5.719942	0.786989
C	-4.016893	-3.160284	-1.722589	H	5.263540	5.470637	-0.507311
C	-5.211066	-2.918511	-1.112183	H	4.714755	5.621632	1.172036

C	-2.859034	-1.614804	-0.231652	H	6.988755	3.987892	2.433942
C	-2.872174	1.611290	-0.227424	H	6.250765	2.384053	2.390772
C	-4.027117	1.665254	0.640244	H	5.285990	3.780499	2.876229
C	-5.234597	2.275200	0.178064	H	6.541383	3.382842	-1.260128
C	-5.235391	2.898779	-1.101601	H	7.725981	3.765882	0.000052
C	-4.043887	3.151672	-1.712706	H	7.013592	2.152108	-0.085527

6-*P*

	X	Y	Z		X	Y	Z
C	4.615184	1.360679	2.496217	C	5.964870	0.518443	-1.883423
C	3.422976	0.675573	2.112496	C	-7.475989	-2.716546	0.453162
C	2.193052	1.235455	2.534885	C	-6.301825	-4.711348	-0.478789
C	4.528713	2.616775	3.139397	C	-6.247009	-4.244430	2.001420
C	2.135035	2.444345	3.195707	C	-6.055067	5.119958	-0.772564
C	3.310502	3.163218	3.473422	C	-6.973920	3.053386	-1.832983
C	3.528696	-0.612788	1.436668	C	-7.102495	3.370333	0.667673
C	4.787927	-1.265145	1.479366	C	3.283465	-3.173060	-3.476316
C	5.960386	-0.551375	1.881230	C	2.114177	-2.444576	-3.197532
C	5.886588	0.733014	2.311791	C	2.182564	-1.236739	-2.535780
C	3.417301	-0.687389	-2.113565	C	4.506329	-2.637140	-3.142241
C	1.233382	-0.651460	0.287342	H	1.275546	0.693035	2.362957
C	1.239484	0.657210	-0.287912	H	5.449901	3.129652	3.396292
C	0.024619	1.382087	-0.304812	H	1.174167	2.835971	3.509270
C	-1.213737	0.710467	-0.103443	H	3.257390	4.121477	3.977778
C	-1.219866	-0.684374	0.097716	H	6.914326	-1.065263	1.829178
C	0.012227	-1.365868	0.303323	H	6.781549	1.277220	2.594569
C	-2.464823	-1.399758	0.172776	H	-5.831491	-0.899697	0.163382
C	-2.451432	1.436795	-0.181576	H	-3.688756	-4.537601	0.663398
C	-3.699158	-0.704444	0.090162	H	-3.646640	4.588319	-0.649422
C	-4.894002	-1.429811	0.214534	H	-5.823128	0.964605	-0.195141
C	-4.916819	-2.805350	0.421998	H	-2.041937	-5.510263	0.226014
C	-3.689499	-3.471604	0.484961	H	0.107405	-6.677371	0.264556
C	-2.468248	-2.810514	0.349436	H	2.202614	-5.434351	0.441749
C	-2.443770	2.845448	-0.350168	H	2.247961	5.431974	-0.435114
C	-3.662071	3.522821	-0.482570	H	0.162619	6.691401	-0.257156
C	-4.889643	2.865854	-0.431032	H	-1.996007	5.542773	-0.222997
C	-4.876541	1.483890	-0.234717	H	3.831706	-4.428824	0.733677
C	-3.694032	0.748005	-0.106878	H	5.842406	-3.127253	1.192982
C	-6.224065	-3.601208	0.595054	H	5.868265	3.095473	-1.195537
C	-6.233539	3.601273	-0.590604	H	3.868560	4.413334	-0.734901
C	0.024457	-2.801206	0.433572	H	6.770494	-1.316704	-2.597678
C	0.048410	2.816738	-0.432542	H	6.923060	1.024409	-1.831852
C	-1.192684	-3.532704	0.378810	H	-7.530697	-2.243875	-0.531412

C	-1.133770	-4.930038	0.315807	H	-7.510959	-1.933158	1.215112
C	0.084322	-5.595208	0.326997	H	-8.372480	-3.330467	0.573761
C	1.271850	-4.885336	0.419326	H	-5.468852	-5.413865	-0.402662
C	1.271201	-3.485612	0.488624	H	-6.286244	-4.283176	-1.484696
C	1.300862	3.491010	-0.486491	H	-7.228559	-5.282200	-0.366033
C	1.312842	4.890315	-0.414304	H	-5.403211	-4.921397	2.152911
C	0.130702	5.609543	-0.321204	H	-6.203390	-3.478325	2.780228
C	-1.092822	4.954812	-0.312050	H	-7.167176	-4.820276	2.140844
C	-1.163530	3.557593	-0.376674	H	-5.557756	5.577295	0.087270
C	2.493376	-2.718279	0.713634	H	-5.479383	5.358824	-1.670880
C	2.516411	2.713674	-0.713163	H	-7.035272	5.593000	-0.875480
C	3.748898	-3.360145	0.871678	H	-7.175257	1.983240	-1.746641
C	4.874455	-2.639654	1.151056	H	-6.382382	3.209244	-2.739114
C	2.426566	-1.312903	0.819057	H	-7.933346	3.564417	-1.959951
C	2.438141	1.308873	-0.819027	H	-7.318472	2.311341	0.826046
C	3.534135	0.599822	-1.437323	H	-6.600239	3.745968	1.563213
C	4.798619	1.241827	-1.480716	H	-8.058857	3.893144	0.568770
C	4.896466	2.615671	-1.152594	H	3.222122	-4.130382	-3.981522
C	3.777004	3.345286	-0.872380	H	1.149944	-2.827826	-3.511140
C	4.603559	-1.382239	-2.498255	H	1.269691	-0.686746	-2.363285
C	5.880213	-0.765205	-2.314253	H	5.423102	-3.157510	-3.399861

6-*M*

	X	Y	Z		X	Y	Z
C	-4.615193	1.360637	2.496207	C	-5.964876	0.518432	-1.883434
C	-3.422980	0.675533	2.112499	C	7.476006	-2.716480	0.453230
C	-2.193063	1.235412	2.534914	C	6.247010	-4.244416	2.001424
C	-4.528732	2.616730	3.139395	C	6.301902	-4.711285	-0.478794
C	-2.135056	2.444298	3.195744	C	6.055021	5.119980	-0.772646
C	-3.310526	3.163171	3.473443	C	7.102459	3.370408	0.667651
C	-3.528692	-0.612826	1.436666	C	6.973902	3.053386	-1.832997
C	-4.787922	-1.265185	1.479344	C	-3.283404	-3.172971	-3.476450
C	-5.960386	-0.551416	1.881195	C	-2.114128	-2.444490	-3.197607
C	-5.886594	0.732971	2.311763	C	-2.182540	-1.236683	-2.535802
C	-3.417293	-0.687365	-2.113592	C	-4.506281	-2.637079	-3.142374
C	-1.233379	-0.651487	0.287336	H	-1.275554	0.692991	2.363002
C	-1.239492	0.657190	-0.287904	H	-5.449925	3.129605	3.396278
C	-0.024633	1.382076	-0.304796	H	-1.174193	2.835921	3.509326
C	1.213729	0.710462	-0.103443	H	-3.257423	4.121426	3.977807
C	1.219869	-0.684382	0.097703	H	-6.914325	-1.065304	1.829131
C	-0.012219	-1.365885	0.303310	H	-6.781560	1.277175	2.594532
C	2.464831	-1.399757	0.172762	H	5.831496	-0.899664	0.163390
C	2.451419	1.436799	-0.181577	H	3.688792	-4.537586	0.663405

C	3.699161	-0.704432	0.090148	H	3.646602	4.588333	-0.649419
C	4.894011	-1.429788	0.214530	H	5.823117	0.964630	-0.195179
C	4.916841	-2.805326	0.421994	H	2.041977	-5.510268	0.226054
C	3.689526	-3.471590	0.484959	H	-0.107354	-6.677392	0.264632
C	2.468268	-2.810511	0.349430	H	-2.202572	-5.434386	0.441816
C	2.443747	2.845453	-0.350160	H	-2.247999	5.431954	-0.435037
C	3.662042	3.522835	-0.482573	H	-0.162662	6.691392	-0.257086
C	4.889619	2.865874	-0.431052	H	1.995970	5.542774	-0.222957
C	4.876528	1.483910	-0.234742	H	-3.83169	-4.428856	0.733635
C	3.694024	0.748017	-0.106893	H	-5.842397	-3.127291	1.192926
C	6.224093	-3.601167	0.595070	H	-5.868301	3.095439	-1.195436
C	6.233508	3.601302	-0.590639	H	-3.868606	4.413303	-0.734761
C	-0.024437	-2.801223	0.433570	H	-6.770470	-1.316691	-2.597784
C	-0.048434	2.816729	-0.432502	H	-6.923071	1.024387	-1.831857
C	1.192710	-3.532712	0.378815	H	8.372504	-3.330382	0.573867
C	1.133807	-4.930047	0.315838	H	7.530746	-2.243810	-0.531343
C	-0.084280	-5.595228	0.327048	H	7.510926	-1.933091	1.215180
C	-1.271813	-4.885364	0.419369	H	7.167183	-4.820248	2.140862
C	-1.271175	-3.485638	0.488636	H	5.403219	-4.921400	2.152878
C	-1.300891	3.490995	-0.486426	H	6.203355	-3.478326	2.780244
C	-1.312877	4.890301	-0.414233	H	5.468942	-5.413820	-0.402702
C	-0.130740	5.609534	-0.321141	H	6.286338	-4.283093	-1.484693
C	1.092787	4.954808	-0.312004	H	7.228645	-5.28212	-0.366027
C	1.163502	3.557591	-0.376642	H	7.035222	5.593030	-0.875567
C	-2.493358	-2.718312	0.713634	H	5.479343	5.358812	-1.670974
C	-2.516440	2.713656	-0.713090	H	5.557698	5.577337	0.087171
C	-3.748884	-3.360179	0.871655	H	8.058814	3.893229	0.568740
C	-4.874445	-2.639691	1.151022	H	7.318447	2.311424	0.826054
C	-2.426554	-1.312937	0.819060	H	6.600191	3.746063	1.563177
C	-2.438160	1.308858	-0.818988	H	6.382367	3.209211	-2.739136
C	-3.534149	0.599817	-1.437301	H	7.933323	3.564424	-1.959975
C	-4.798637	1.241813	-1.480683	H	7.175250	1.983245	-1.746623
C	-4.896498	2.615644	-1.152510	H	-3.222042	-4.130269	-3.981700
C	-3.777042	3.345261	-0.872275	H	-1.149884	-2.827717	-3.511209
C	-4.603535	-1.382209	-2.498332	H	-1.269675	-0.686691	-2.363256
C	-5.880199	-0.765196	-2.314320	H	-5.423043	-3.157448	-3.400034

6-TS

	X	Y	Z		X	Y	Z
C	5.007485	-1.766918	1.785246	C	6.030175	2.051843	-0.381293
C	3.718234	-1.593780	1.196512	C	-7.187769	-2.849593	1.511185
C	2.597308	-1.615031	2.059584	C	-6.623672	-4.476705	-0.296453
C	5.124620	-1.894668	3.187064	C	-5.605150	-4.718295	2.000245

C	2.737009	-1.781922	3.420523	C	-5.929914	5.212141	0.720263
C	4.011602	-1.910095	3.996845	C	-7.303983	3.217941	0.110788
C	3.591250	-1.629433	-0.253472	C	-6.327164	3.435072	2.428377
C	4.720971	-2.063547	-0.998436	C	4.020666	1.887721	4.004353
C	6.015941	-2.075432	-0.391100	C	2.745836	1.768715	3.426546
C	6.158596	-1.906272	0.948003	C	2.606335	1.606299	2.065066
C	3.727872	1.580585	1.202871	C	5.134308	1.867415	3.195537
C	1.151620	-0.719282	-0.643849	H	1.603888	-1.532712	1.641575
C	1.157015	0.729675	-0.641954	H	6.115017	-2.014913	3.614105
C	-0.101599	1.388933	-0.727918	H	1.853516	-1.816018	4.048089
C	-1.303586	0.712382	-0.419160	H	4.115458	-2.033846	5.068933
C	-1.308669	-0.684653	-0.421103	H	6.875123	-2.290522	-1.017747
C	-0.111735	-1.369094	-0.731093	H	7.137466	-1.959610	1.412482
C	-2.535360	-1.398187	-0.213652	H	-5.676055	-0.967828	1.005893
C	-2.524641	1.434255	-0.211156	H	-3.759798	-4.567744	-0.252981
C	-3.685624	-0.714899	0.254155	H	-3.727498	4.611114	-0.249229
C	-4.807469	-1.476256	0.615850	H	-5.675990	1.021779	0.990355
C	-4.843372	-2.864070	0.480623	H	-2.540894	-5.136830	-1.886987
C	-3.734700	-3.496897	-0.097848	H	-0.690603	-5.998897	-3.250196
C	-2.591369	-2.791133	-0.470413	H	1.443179	-4.797778	-3.377562
C	-2.572615	2.824693	-0.465996	H	1.478411	4.816718	-3.359725
C	-3.715743	3.541897	-0.095924	H	-0.646685	6.032677	-3.227241
C	-4.826084	2.914831	0.473782	H	-2.503535	5.178541	-1.868827
C	-4.796790	1.521966	0.607706	H	3.130578	-3.594518	-3.559782
C	-3.682427	0.754366	0.254441	H	5.397581	-2.907024	-2.875211
C	-6.054975	-3.707962	0.918940	H	5.419279	2.895440	-2.863365
C	-6.077385	3.693134	0.924106	H	3.157485	3.601176	-3.546771
C	-0.242078	-2.691576	-1.286767	H	7.149206	1.923499	1.423009
C	-0.222840	2.713397	-1.278917	H	6.891351	2.262966	-1.006552
C	-1.472352	-3.393875	-1.203525	H	-8.015509	-3.495598	1.815372
C	-1.603897	-4.595264	-1.910284	H	-6.859760	-2.297169	2.395953
C	-0.562837	-5.075557	-2.696502	H	-7.580950	-2.134257	0.783443
C	0.639791	-4.384893	-2.781777	H	-7.485265	-5.079471	0.006625
C	0.829880	-3.195778	-2.066418	H	-5.885375	-5.151987	-0.734627
C	0.853300	3.213641	-2.055694	H	-6.949603	-3.785320	-1.078198
C	0.671922	4.406846	-2.766044	H	-4.827948	-5.391179	1.630902
C	-0.526009	5.105773	-2.677903	H	-5.207577	-4.199000	2.876385
C	-1.570840	4.629811	-1.894502	H	-6.452398	-5.331305	2.322867
C	-1.449059	3.423421	-1.193557	H	-6.834107	5.716857	1.070762
C	2.136134	-2.555545	-1.938811	H	-5.085624	5.618381	1.283715
C	2.154851	2.563691	-1.929887	H	-5.797688	5.471336	-0.333677
C	3.270262	-2.987110	-2.674622	H	-8.200449	3.761704	0.424364
C	4.532188	-2.628752	-2.283849	H	-7.496550	2.151702	0.248859
C	2.315800	-1.588207	-0.922746	H	-7.155103	3.394601	-0.957896
C	2.327245	1.591212	-0.917454	H	-5.474999	3.767228	3.027335
C	3.602523	1.621481	-0.247114	H	-7.213734	3.981157	2.765161
C	4.735690	2.050418	-0.989749	H	-6.489391	2.375467	2.638259
C	4.551616	2.621213	-2.273437	H	4.124300	2.008162	5.076841
C	3.292466	2.989770	-2.663629	H	1.861993	1.806564	4.053409

C	5.017658	1.743984	1.793272	H	1.612793	1.531136	1.646014
C	6.170443	1.877991	0.957471	H	6.125061	1.980306	3.623754

9-*P*

	X	Y	Z		X	Y	Z
C	1.582715	-0.726052	0.03446	C	-2.977498	-1.728952	-1.832463
C	1.581322	0.729023	-0.034398	C	-2.980829	1.723322	1.832430
C	0.441041	-1.587530	-0.213146	C	-1.770990	0.122737	-2.769858
C	0.437990	1.588322	0.213148	C	-1.770741	-0.125899	2.770076
C	-0.697653	-1.236083	-1.049088	C	-2.946860	-0.643171	-2.676454
C	-0.700028	1.234769	1.049128	C	-2.948097	0.637708	2.676555
C	-0.680923	-0.166737	-1.977509	H	0.217632	0.420805	-2.096460
C	-0.681229	0.165589	1.977701	H	0.218467	-0.420183	2.096754
C	2.819465	-1.358123	0.351524	H	-1.714038	0.943534	-3.477493
C	2.816859	1.363451	-0.351484	H	-1.712200	-0.946487	3.477825
C	4.042033	-0.639109	0.222955	H	-3.808232	-0.399063	-3.289890
C	4.040802	0.646785	-0.222891	H	-3.809000	0.392009	3.290014
C	2.835731	-2.715376	0.780907	H	-3.857638	-2.363885	-1.786752
C	2.830524	2.720710	-0.780938	H	-3.862195	2.356547	1.786635
C	1.679470	-3.429791	0.858279	H	-2.743147	-3.900845	-0.279516
C	1.672888	3.432889	-0.858386	H	-2.750651	3.895500	0.279264
C	0.469330	-2.899004	0.327354	H	-0.667049	-4.681629	0.796705
C	0.463762	2.899806	-0.327455	H	-0.676043	4.680197	-0.796979
C	-0.706662	-3.711456	0.310128	H	1.677069	-4.441860	1.251602
C	-0.713794	3.709991	-0.310321	H	1.668547	4.444925	-1.251778
C	-1.847208	-3.287361	-0.293035	H	3.783470	-3.156153	1.074983
C	-1.853532	3.283747	0.292853	H	3.777416	3.163289	-1.075030
C	-1.857642	-2.069848	-1.038327	H	4.974042	-1.157531	0.426566
C	-1.861625	2.066290	1.038265	H	4.971816	1.166987	-0.426512

9-*M*

	X	Y	Z		X	Y	Z
C	-1.584671	-0.721877	0.034547	C	2.972795	-1.736751	-1.832556
C	-1.579362	0.733191	-0.034310	C	2.985488	1.715303	1.832423
C	-0.445318	-1.586396	-0.213150	C	1.771283	0.118383	-2.769561
C	-0.433697	1.589410	0.213161	C	1.770338	-0.130426	2.770393
C	0.694308	-1.237879	-1.049048	C	2.945069	-0.650739	-2.676346
C	0.703353	1.232872	1.049204	C	2.949788	0.629927	2.676727
C	0.680460	-0.168292	-1.977239	H	-0.216487	0.421737	-2.096027
C	0.681630	0.163916	1.977976	H	-0.219663	-0.419363	2.097154
C	-2.823114	-1.350640	0.351564	H	1.716542	0.939476	-3.477024
C	-2.813184	1.370935	-0.351409	H	1.709549	-0.950721	3.478288

C	-4.043739	-0.628324	0.223033	H	3.807084	-0.408846	-3.289754
C	-4.039050	0.657568	-0.222787	H	3.810016	0.381973	3.290222
C	-2.843015	-2.707885	0.780828	H	3.851229	-2.374049	-1.786978
C	-2.823173	2.728201	-0.780947	H	3.868591	2.346092	1.786525
C	-1.688686	-3.425426	0.858082	H	2.732614	-3.908259	-0.279918
C	-1.663614	3.437225	-0.858491	H	2.761208	3.887899	0.278956
C	-0.477130	-2.897854	0.327176	H	0.654473	-4.683595	0.796242
C	-0.455920	2.900907	-0.327559	H	0.688735	4.678133	-0.797294
C	0.696672	-3.713467	0.309798	H	-1.689015	-4.437536	1.251301
C	0.723843	3.707880	-0.310536	H	-1.656545	4.449207	-1.251977
C	1.838335	-3.292362	-0.293340	H	-3.791925	-3.146149	1.074877
C	1.862428	3.278592	0.292644	H	-3.768861	3.173334	-1.075057
C	1.852042	-2.074767	-1.038451	H	-4.977143	-1.144235	0.426618
C	1.867219	2.061214	1.038215	H	-4.968666	1.18026	-0.426412

10-*P*

	X	Y	Z		X	Y	Z
C	0.440002	1.720456	-0.577414	C	-3.691673	0.597533	-1.514832
C	-0.440269	1.720419	0.57742	C	3.687464	-1.801574	1.453942
C	0.757757	0.553698	-1.381462	C	2.313577	0.61391	1.540244
C	-0.757832	0.553628	1.381522	C	3.691645	0.597935	1.514824
C	-0.096962	-0.603617	-1.566641	C	4.391179	-0.619481	1.437533
C	0.097045	-0.603590	1.566650	C	-3.68724	-1.801981	-1.454013
C	-1.551917	-0.579986	-1.495409	C	-4.391066	-0.619957	-1.437583
C	1.552000	-0.579791	1.495355	C	-2.313601	0.613642	-1.540310
C	1.054824	2.952473	-0.931694	H	4.237446	1.534643	1.564368
C	-1.055255	2.952354	0.931676	H	1.807638	1.563977	1.632055
C	0.540730	4.177358	-0.413148	H	4.212491	-2.752614	1.450101
C	-0.541364	4.177297	0.413065	H	5.475953	-0.625653	1.401043
C	2.191043	2.957599	-1.790662	H	-5.475845	-0.626254	-1.401193
C	-2.19144	2.957337	1.790687	H	-1.807738	1.563746	-1.632170
C	2.709094	1.785552	-2.250718	H	-4.237563	1.534184	-1.564387
C	-2.709298	1.785236	2.250808	H	-4.212182	-2.753076	-1.450252
C	2.005474	0.560831	-2.065836	H	-2.131414	-3.970906	-1.707128
C	-2.005526	0.560604	2.065936	H	2.131899	-3.970650	1.707134
C	2.542708	-0.654122	-2.576316	H	0.276990	-3.954277	-2.232985
C	-2.542583	-0.654418	2.576434	H	-0.276510	-3.954308	2.232967
C	1.857013	-1.822298	-2.441925	H	2.294226	-2.762876	-2.762868
C	-1.856754	-1.822507	2.441999	H	-2.293851	-2.763140	2.762937
C	0.510734	-1.816886	-1.97854	H	3.524586	-0.634706	-3.039230
C	-0.510498	-1.816935	1.978553	H	-3.524440	-0.635135	3.039402
C	-0.242529	-3.031886	-1.990868	H	3.635186	1.771422	-2.817357
C	0.242901	-3.031854	1.990857	H	-3.635368	1.770994	2.817482

C	-2.274438	-1.812075	-1.522392	H	2.668739	3.905865	-2.018052
C	2.274655	-1.811814	1.522328	H	-2.669268	3.905541	2.018066
C	-1.571320	-3.040453	-1.712247	H	0.996695	5.109556	-0.732856
C	1.571685	-3.040266	1.712209	H	-0.997474	5.109443	0.732714

10-*M*

	X	Y	Z		X	Y	Z
C	-2.709564	1.785374	-2.250229	C	3.687076	-1.801868	-1.454258
C	-2.191482	2.957444	-1.790315	C	2.313373	0.613712	-1.540575
C	-2.005855	0.560670	-2.065480	C	4.390878	-0.619829	-1.437985
C	-1.055090	2.952390	-0.931551	C	3.691442	0.597636	-1.515215
C	-0.758016	0.553613	-1.381352	C	-3.687142	-1.801631	1.454097
C	-0.440178	1.720423	-0.577340	C	-2.313335	0.613894	1.540323
C	-2.543076	-0.654297	-2.575880	C	-4.390888	-0.619558	1.437651
C	-1.857276	-1.822433	-2.441592	C	-3.691403	0.597879	1.514832
C	-0.510953	-1.816946	-1.978411	H	-4.212154	-2.752682	1.450353
C	0.096741	-0.603618	-1.566665	H	-5.475667	-0.625768	1.401223
C	0.242363	-3.031917	-1.990705	H	-4.237243	1.534567	1.564344
C	1.551726	-0.579936	-1.495561	H	-1.807412	1.563972	1.632102
C	2.274269	-1.812003	-1.522500	H	-3.635765	1.771173	-2.816697
C	1.571171	-3.040417	-1.712200	H	-2.669255	3.905691	-2.017633
C	0.541226	4.177300	0.412971	H	-3.525019	-0.634967	-3.038669
C	-0.540989	4.177306	-0.413087	H	-2.294498	-2.763038	-2.762456
C	1.055262	2.952386	0.931502	H	-0.277168	-3.954363	-2.232603
C	0.440304	1.720429	0.577336	H	2.131289	-3.970859	-1.706987
C	2.191627	2.957431	1.790303	H	0.997328	5.109471	0.732573
C	2.709628	1.785362	2.250311	H	-0.997051	5.109482	-0.732728
C	2.005862	0.560683	2.065609	H	2.669437	3.905667	2.017587
C	0.758057	0.553646	1.381419	H	3.635803	1.771148	2.816820
C	2.542993	-0.654278	2.576120	H	3.524911	-0.634967	3.038960
C	-0.096763	-0.603535	1.566742	H	2.294293	-2.762992	2.762832
C	0.510843	-1.816864	1.978614	H	0.276937	-3.954252	2.232945
C	1.857137	-1.822387	2.441879	H	-2.131480	-3.970673	1.707144
C	-0.242532	-3.031798	1.990941	H	4.212044	-2.752943	-1.450481
C	-1.551740	-0.579794	1.495499	H	5.475660	-0.626086	-1.401660
C	-1.571318	-3.040257	1.712333	H	4.237322	1.534292	-1.564871
C	-2.274343	-1.811825	1.522472	H	1.807485	1.563804	-1.632398

Reference

[1] Qiu, Z.; Asako, S.; Hu, Y.; Ju, C. W.; Liu, T.; Rondin, L.; Schollmeyer, D.; Lauret, J. S.; Müllen, K.; Narita, A. *J. Am. Chem. Soc.* **2020**, *142*, 14814-14819.

[2] Birks, J. B.; Birch, D. J. S.; Cordemans, E.; Vander Donckt, E. *Chem. Phys. Lett.* **1975**, *43*, 33-36.

[3]. Vander Donckt, E.; Nasielski, J.; Greenleaf, J. R.; Birks, J. B. *Chem. Phys. Lett.* **1968**, *2*, 409-410.

[4]. Nakai, Y.; Mori, T.; Inoue, Y. *J. Phys. Chem. A.* **2012**, *116*, 7372−7385.

[5]. Martin, R. H.; Marchant, M. J. *Tetrahedron* **1974**, *30*, 343−345.

[6] Gaussian 09, Revision D.01, Frisch, M. J.; Trucks, G. W.; Schlegel, H. B.; Scuseria, G. E.; Robb, M. A.; Cheeseman, J. R.; Scalmani, G.; Barone, V.; Mennucci, B.; Petersson, G. A.; Nakatsuji, H.; Caricato, M.; Li, X.; Hratchian, H. P.; Izmaylov, A. F.; Bloino, J.; Zheng, G.; Sonnenberg, J. L.; Hada, M.; Ehara, M.; Toyota, K.; Fukuda, R.; Hasegawa, J.; Ishida, M.; Nakajima, T.; Honda, Y.; Kitao, O.; Nakai, H.; Vreven, T.; Montgomery, Jr., J. A.; Peralta, J. E.; Ogliaro, F.; Bearpark, M.; Heyd, J. J.; Brothers, E.; Kudin, K. N.; Staroverov, V. N.; Kobayashi, R.; Normand, J.; Raghavachari, K.; Rendell, A.; Burant, J. C.; Iyengar, S. S.; Tomasi, J.; Cossi, M.;Rega, N.; Millam, N. J.; Klene, M.; Knox, J. E.; Cross, J. B.; Bakken, V.; Adamo, C.; Jaramillo, J.; Gomperts, R.; Stratmann, R. E.; Yazyev, O.; Austin, A. J.; Cammi, R.; Pomelli, C.; Ochterski, J. W.; Martin, R. L.; Morokuma, K.; Zakrzewski, V. G.; Voth, G. A.; Salvador, P.; Dannenberg, J. J.; Dapprich, S.; Daniels, A. D.; Farkas, Ö.; Foresman, J. B.; Ortiz, J. V.; Cioslowski, J.; Fox, D. J. Gaussian, Inc., Wallingford CT, 2013.

[7]. Lu, T.; Chen, F., J. *Comput. Chem.* **2012**, *33*, 580-592.
